# Metagenomic comparison of gut communities between wild and captive Himalayan griffons

**DOI:** 10.3389/fvets.2024.1403932

**Published:** 2024-05-09

**Authors:** You Wang, Jundie Zhai, Boyu Tang, Yonggang Dong, Shengzhen Sun, Shunfu He, Wenxin Zhao, Zhuoma Lancuo, Qiangqiang Jia, Wen Wang

**Affiliations:** ^1^State Key Laboratory of Plateau Ecology and Agriculture, Qinghai University, Xining, Qinghai, China; ^2^College of Eco-Environmental Engineering, Qinghai University, Xining, Qinghai, China; ^3^Animal Disease Prevention and Control Center of Qinghai Province, Xining, Qinghai, China; ^4^Xining Wildlife Park of Qinghai Province, Xining, Qinghai, China; ^5^College of Finance and Economics, Qinghai University, Xining, Qinghai, China

**Keywords:** gut microbiome, metagenome, metagenome-assembled genomes, conservation, scavenger

## Abstract

**Introduction:**

Himalayan griffons (*Gyps himalayensis*), known as the scavenger of nature, are large scavenging raptors widely distributed on the Qinghai-Tibetan Plateau and play an important role in maintaining the balance of the plateau ecosystem. The gut microbiome is essential for host health, helping to maintain homeostasis, improving digestive efficiency, and promoting the development of the immune system. Changes in environment and diet can affect the composition and function of gut microbiota, ultimately impacting the host health and adaptation. Captive rearing is considered to be a way to protect Himalayan griffons and increase their population size. However, the effects of captivity on the structure and function of the gut microbial communities of Himalayan griffons are poorly understood. Still, availability of sequenced metagenomes and functional information for most griffons gut microbes remains limited.

**Methods:**

In this study, metagenome sequencing was used to analyze the composition and functional structures of the gut microbiota of Himalayan griffons under wild and captive conditions.

**Results:**

Our results showed no significant differences in the alpha diversity between the two groups, but significant differences in beta diversity. Taxonomic classification revealed that the most abundant phyla in the gut of Himalayan griffons were *Fusobacteriota*, *Proteobacteria*, *Firmicutes_A*, *Bacteroidota*, *Firmicutes*, *Actinobacteriota*, and *Campylobacterota*. At the functional level, a series of Kyoto Encyclopedia of Genes and Genome (KEGG) functional pathways, carbohydrate-active enzymes (CAZymes) categories, virulence factor genes (VFGs), and pathogen-host interactions (PHI) were annotated and compared between the two groups. In addition, we recovered nearly 130 metagenome-assembled genomes (MAGs).

**Discussion:**

In summary, the present study provided a first inventory of the microbial genes and metagenome-assembled genomes related to the Himalayan griffons, marking a crucial first step toward a wider investigation of the scavengers microbiomes with the ultimate goal to contribute to the conservation and management strategies for this near threatened bird.

## 1 Introduction

The gastrointestinal tracts of animals contain numerous and variable microbial communities (including viruses, bacteria, archaea, and eukaryotes such as protists and fungi), which were previously considered as pathogens and harmful to the host. But now we have to change this view and admit that these symbiotic microbial communities (microbiota) together with their genomes (microbiome) play an important role in maintaining the host health in many ways, such as digestion of food, absorption of nutrients, modulation of intestinal immunity and homeostasis, inhibiting of pathogen growth, toxin metabolism, and overall regulation of host physiological functions (1). This is driven by the development of high-throughput sequencing techniques, which enabled us to obtain greater insights into the taxonomy information (e.g., by the 16S rRNA gene analyses), and functional roles (e.g., by the sequencing of metagenomes) of the gut microbiome (2). Despite the extensive research highlighting the benefits and significance of the gut microbiome, one neglected field of study is wildlife (3). Currently, the majority of research on the gut microbiome is concentrated on humans, laboratory animals, model organisms, and domesticated animals with economic value (4). It is becoming clear that characterizing gut microbiomes associated with wildlife has important implications for understanding the physiology (5), ecology (6), evolution (7), conservation management (8), and zoonotic disease (9) of wild animals. Levin et al. (10) reported a large-scale, annotated metagenomic database of the gut microbiota of 184 unique wildlife species, and found that 75% of the constructed bacterial species were unknown, which indicated that the wildlife gut microbiome is a valuable yet largely untapped gold mine for the discovery of novel biological functions and technologies (11).

Birds are found all over the world and are the most diverse group of amniotic vertebrates with more than 11,000 species, each with their own unique appearance and habits. Birds represent ideal research systems for studying the roles of gut microbiome, due to their strong geographical dispersal ability, the wide distribution range, contact with many intermediate organisms, extremely complex and unique diets (e.g., fruit, seeds, insects, carrion, and small animals), physiological traits (e.g., high energy consumption for the flight), and extreme morphological diversity (12). Additionally, many bird species undergo lengthy seasonal migrations spanning long distances, hence the resulting shifts in food and living environment forming strong selective pressure on gut microbiome (13). In the past 2 decades, the studies of gut microbial composition, diversity, and function related to birds were markedly increased (14). According to the statistics, more than 200 papers on “bird gut microbiome” had been published in the year 2021 (15). Waite and Taylor conducted the first meta-analysis of the bird gut microbiota, and found that much like other animal hosts, the gut microbiota of bird mainly consisted of members in the *Firmicutes*, *Actinobacteria*, *Bacteroidetes*, and *Proteobacteria*, with the relative proportions varied substantially in different bird ecology groups (16). Fluctuations in the bird gut microbiome are often linked to the complex interaction determined by the host genetic background (17), diet (18), geographic location (19), age (20), gender (21), lifestyle (22), social class of the host (23), and the climate of the habitat (24). Therefore, understanding the relative importance of these factors has become a central theme in bird gut microbiome (15). Song et al. (25) evaluated contributions of diet, phylogeny, and physiology to building gut microbiomes by using microbiome data from about 900 vertebrate species (including 315 mammals, 491 birds, and other animals). They found that compared with that non-flying mammals, the bird gut microbiomes had a weak relationship with the diet or host phylogeny. However, more findings of these studies on the influencing factors were mixed. For example, some studies have concluded that the influence of host factors is greater than that of environmental factors (26), while other studies have shown that living environment is the main driving factor for the establishment of gut microbiota (27). This suggests that we need more rigorously controlled experiments and more bird species in the future to unravel these specific factors affecting the avian gut microbiota.

The research on the impact of captivity or the artificial rearing environment on the gut microbiota of birds is also a hot field in recent years; especially the captive breeding has become an effective means to protect threatened or endangered wild birds (15). In this context, several wild and matching captive bird species were compared to examine the effects of captivity on bird gut microbiomes, such as the oriental white storks (*Ciconia boyciana*) (28), raptors of seven different species (29), red-crowned cranes (30), and bar-headed geese (31). For most wild animals, captive environments (e.g., rehabilitation, artificial breeding, and zoos, etc.) represent a significant change from the wild. These unnatural conditions can destroy the diversity, composition, and function of gut microbiomes of wild animals. The changes in gut microbiome related to the transition to captivity have been shown to be driven by a variety of different factors, such as the changes or restrictions in diet, antibiotic treatments, reduced exposure to various microbes come from different habitat types, and increased contact with human associated microbes (32). Understanding the broad effects of captivity on the gut microbiome is critical to maintaining the health of captive animals (33). For example, Martínez-Mota et al. (34) highlighted the importance of supplementing artificial food with natural food to promote preservation of the native gut microbiota of captive animals. Thus, comparative analyses of gut microbiomes in the captive versus wild state is helpful to protect biodiversity through the captive breeding of endangered wild birds.

Vultures are unique large raptors at the top of the trophic chain, and feed mainly on carcass from other animals, thus playing an important role in the ecosystem by mitigating the spread of infectious diseases from these carcasses. Currently, there are 23 species of vultures in the world, 16 of them are at risk of extinction (35). Most of the vulture species are declining mainly due to a multiple of threats from anthropogenic activities, such as the illegal use of poisons (36). Vultures constitute a major conservation challenge for the 21st Century (37). It is of great significance to strengthen the monitoring and research of vultures for their protection. Vultures provide a unique model system for studying the mechanism of how they protect themselves against the toxins and pathogens present in the carcass. Until now, the whole genome of the Himalayan griffons (*Gyps himalayensis*) (38), the Bearded vulture (*Gypaetus barbatus*) (38), the Turkey vulture (*Cathartes aura*) (39, 40), and the Cinereous vulture (*Aegypius monachus*) (41) have been sequenced, and these valuable genomic resources provide important insights into the adaptive and protective mechanisms by which these vultures adapted to their scavenging diets. By contrast, research on the gut microbiome (considered as the second genome) of vultures has lagged behind. Up to date, only the Black vulture (*Coragyps atratus*) and the Turkey vulture (*Cathartes aura*) metagenome data have been published. Metagenomic analyses of these two vulture species revealed the importance of microbiome-mediated health protection in adaptation to their unique scavenging diet (42, 43).

Himalayan griffon (*Gyps himalayensis*) is one of the three vulture species (the other two vulture species are *Gypaetus barbatus*, *Aegypius monachus*) that distributed in the Qinghai-Tibetan Plateau, which has the first largest population among these three species. Himalayan griffons feed on the rotting plateau domestic animals (e.g., yaks, Tibetan sheep, Tibetan dogs, horses, etc.) and other plateau wildlife carcasses, playing an important ecological function in removing these carcasses, which may be the source of diseases. Himalayan griffons are currently ranked as “Near Threatened” on the International Union for Conservation of Nature’s (IUCN) Red List, but as the populations continue to decline, they may enter a status of “Vulnerable” in the future. In China, Himalayan griffons belong to the second class of national protected birds. However, little is known about the gut microbiome of Himalayan griffons. Our previous study only used 16S rDNA to reveal the microbial composition of Himalayan griffons, which was limited by the lack of functional resolution (44). Artificial breeding is one of the measures taken to protect Himalayan griffons. Xining Wildlife Park (Qinghai Province, China) has the only artificial breeding population of Himalayan vultures in China. However, there is still lack of information on changes of gut microbiomes of Himalayan griffons in the captive vs. wild state.

Thus, this study presents the first comparative metagenomic survey of gut microbiomes of Himalayan griffons living in the wild and captive environments. Results of the study will facilitate understanding of the impact of captivity on griffons gut microbiome, with the ultimate goal of contributing to the conservation and management strategies for this near threatened bird species.

## 2 Materials and methods

### 2.1 Ethics statement

This study conformed to the guidelines for the care and use of experimental animals established by the Ministry of Science and Technology of the People’s Republic of China (Approval number: 2006-398). The research protocol was reviewed and approved by the Ethical Committee of Qinghai University. This study did not involve capture or any direct manipulation or disturbance of Himalayan griffons.

### 2.2 Sample collection of Himalayan griffons

**Figure 1:**
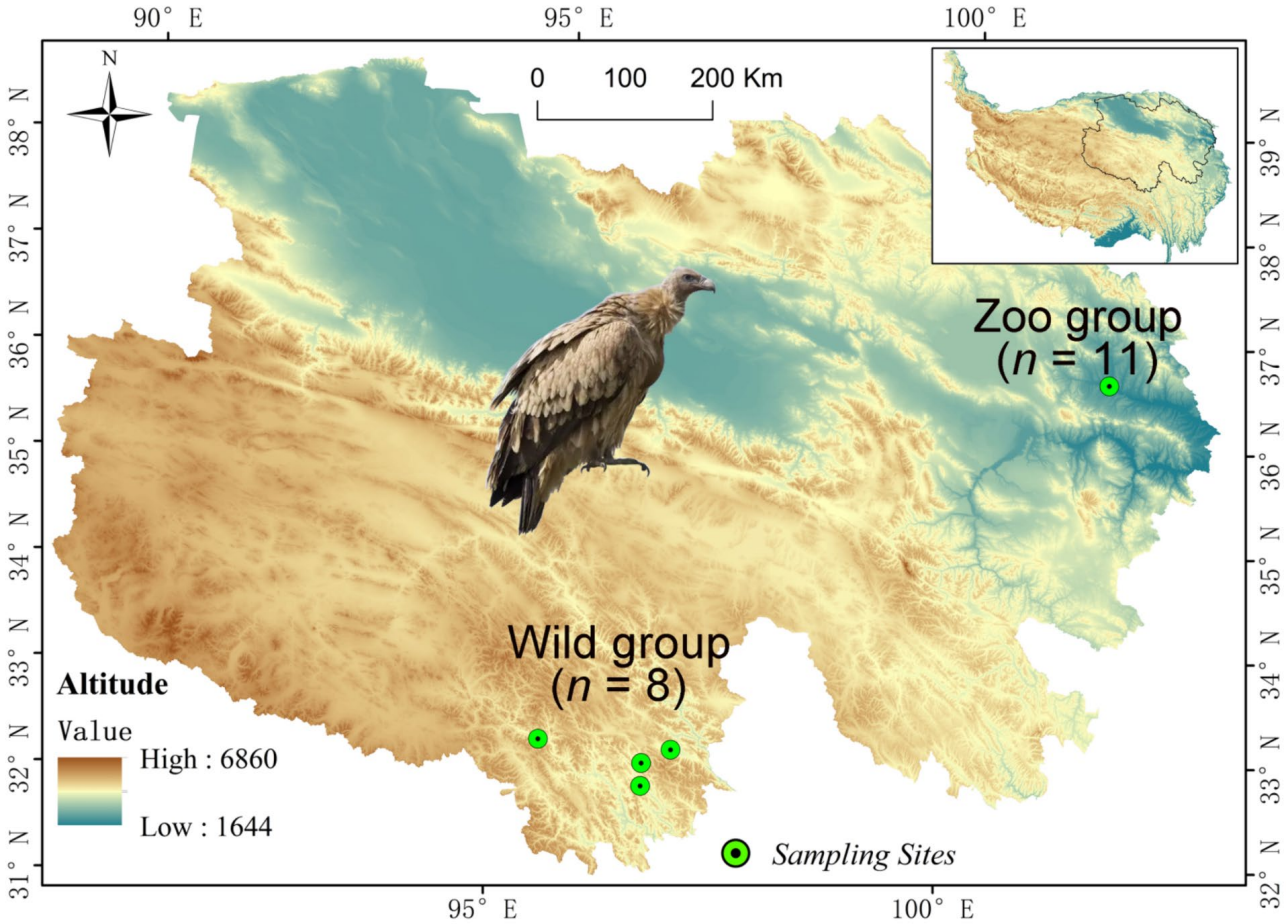
Map of the Qinghai-Tibetan Plateau with sampling sites.

A total of 19 fresh fecal samples of Himalayan griffons were collected from both wild and captive populations (Figure 1). Among them, eight wild fecal samples (Wild group) were randomly selected during the field survey of Himalayan griffons in Yushu City, Qinghai Province, China (Figure 1). A total of 11 fecal samples (Zoo group) were opportunistically collected from captive populations reared in Xining Wildlife Park, China (Figure 1). All the feces were sampled immediately after defecation, and were immediately frozen using liquid nitrogen and then stored at −80°C until use.

### 2.3 DNA library construction and metagenomic sequencing

Genomic DNA was isolated from all fecal samples using the Qiagen QIAamp DNA Stool Mini Kit (Qiagen, Germany) according to the manufacturer’s protocols. DNase-free RNase was used to eliminate any potential RNA contamination in the extracts. DNA concentrations were measured on the Qubit 2.0 fluorimeter (Invitrogen, United States). DNA purity was evaluated using Nanodrop (Thermo Scientific, United States) by calculating 260/280 and 260/230 absorbance ratios. Paired-end (PE) library with insert size of 350 bp for each sample was constructed, followed by a high-throughput sequencing using BGISEQ-500 sequencer with PE reads of length 2 × 150 bp.

Default parameters were used for all bioinformatics software and tools used in this study, unless otherwise stated. High-quality reads were obtained by filtering low-quality reads with ambiguous “N” bases, adapters, and host DNA (Himalayan griffons reference genome GWHBAOP00000000, http://bigd.big.ac.cn/gwh) contamination from the raw reads using Trimmomatic (v.0.39) (45) and Bowtie2 (v.2.4.1) (46) within KneadData pipeline.^1^ The assembly of single sample were performed using MEGAHIT (v.1.2.9) (47) with the optional parameter “-k-list 21, 29, 39, 49, 59, 69, 79, 89, 99, 109, 119, 129, 141.” Then, the assembled contigs with more than 500 bp in length were used for gene prediction by Prodigal (v.2.6) (48) software. A non-redundant microbial gene catalog was constructed by clustering predicted genes using CD-HIT (> 95% sequence identity) (v.4.8.1) (49). Gene coverage of each sample was calculated using BBMap (v.38.57; https://github.com/BioInfoTools/BBMap) with the non-redundant gene catalog as the reference. Taxonomic assignments were performed using the Kraken2 and Bracken methods (50). Based on the taxonomic profile, the alpha diversity was calculated to evaluate the species richness of samples. The nonparametric multivariate statistical analysis methods based on the Bray–Curtis dissimilarity matrix were performed to test the microbial communities. Functional annotations were conducted by aligning the putative amino acid sequences against the KEGG (51), the CAZy database,^2^ VFDB database,^3^ and PHI-base^4^ using DIAMOND with *e*-values ≤1e−5. (v.0.9.22) (52).

### 2.4 Genome binning and analyses

Metagenomic binning of single-sample assembly was conducted using three methods with default parameters: MaxBin2 (v.2.2.5) (53), MetaBAT2 (v.2.12.1) (54), and CONCOCT (v.0.5.0) (55). DAS Tool (v.1.1.0) (56) was used to integrate the Metagenome-assembled genomes (MAGs) obtained from the above methods. All bins were then subjected to RefineM (v.0.0.24) (57) for further refinement. CheckM (v.1.0.12) (58) was then performed to evaluate the completeness and contamination of the bins. The output MAGs were dereplicated at default threshold of 99% average nucleotide identity (ANI) using dRep (v.3.4.2) (59). The taxonomic classifications of the MAGs were inferred using GTDB-Tk (v.2.3.0) (60). The abundance of each MAG in each sample was performed with CoverM (v.0.6.1, https://github.com/wwood/CoverM). The genes of MAGs were predicted and translated to amino acid sequence by Prodigal (v.2.6) (48). For genome annotation, all predicted proteins of MAGs were then functionally characterized using the publicly available databases. All phylogenetic trees of the MAGs were built by PhyloPhlAn (v.3.0.51) (61) and visualized using iTOL (v.5.6.2) (62).

### 2.5 Statistical analysis

All statistics were performed using R software version 4.3.1. Statistical comparisons were performed using nonparametric Wilcoxon tests between the wild and zoo groups. The multiple test correction was conducted using Bonferroni correction. The linear discriminant analysis effect size (LEfSe) was used to compare the groups for significant difference in features (63). Differentially enriched functional pathways were identified using STAMP (64). For all statistical tests, a *p* value of less than 0.05 was considered statistically significant.

## 3 Results

### 3.1 Metagenomic sequencing data

A total of 475,258,034 raw paired-end reads were produced from 19 fecal samples of Himalayan griffons (Supplementary Table S1). After quality control and host removal, a total of 360,209,565 high quality reads were obtained for subsequent analysis with an average length of 295.88 bp (Supplementary Table S1). Each sample contained approximately 18,958,398 reads on average, ranging from 6,110,595 to 29,745,735 reads (Supplementary Table S1). The *de novo* assembly of these high quality reads generated a total of 1.89 Gb of contigs (a total number of 1,274,995 contigs, the longest contig 750,505 bp) (Supplementary Table S2). Gene prediction resulted in a total of 4,869,648 genes with an average length of 482.64 bp (Supplementary Table S3).

### 3.2 Microbial composition analysis

To characterize differences in gut microbial composition, the gut microbiomes of wild and captive Himalayan griffons were compared. A total of 72 phyla (Supplementary Table S4), 2,829 genera (Supplementary Table S5), and 8,165 species (Supplementary Table S6) were identified in the fecal samples by using the k-mer based program Kraken2.

**Figure 2:**
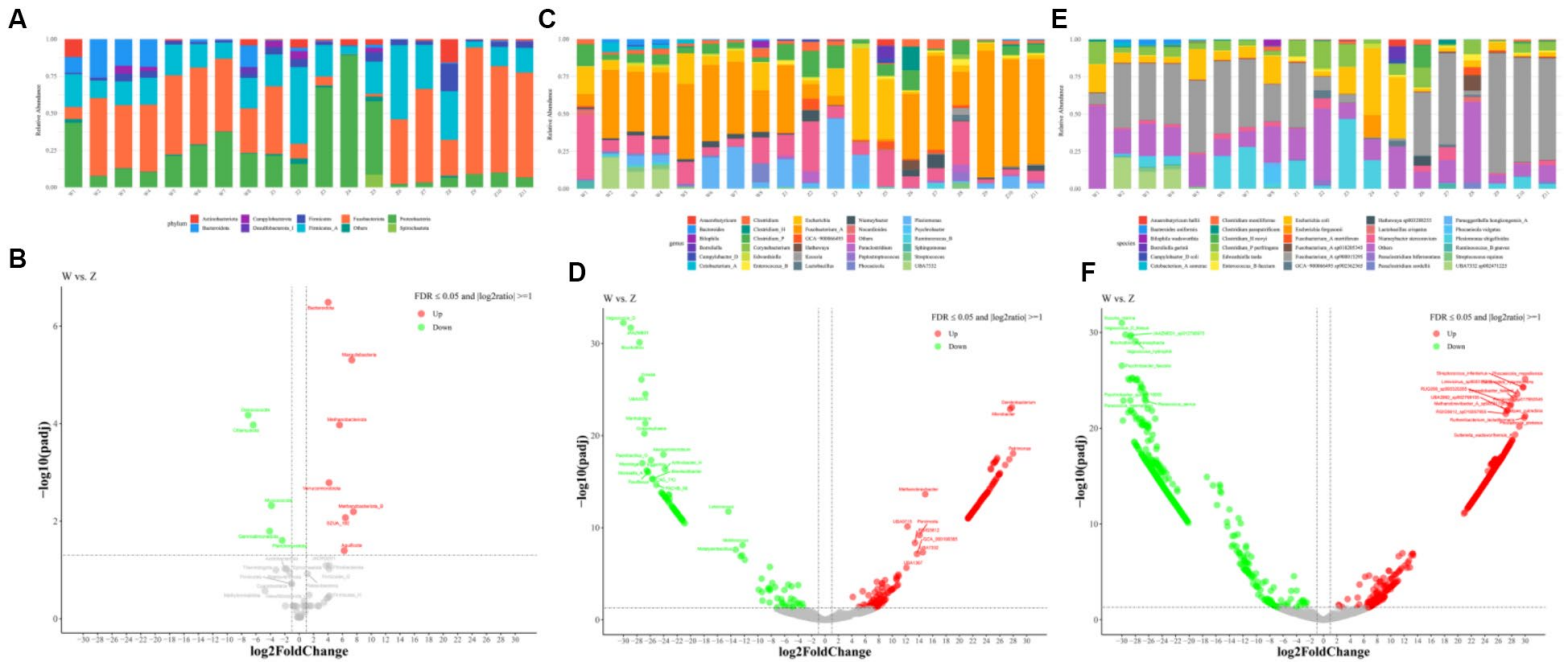
Gut bacterial composition of Himalayan griffons. Relative abundances of dominant phyla **(A)**, genera **(C)**, and species **(E)** in the samples, and the significant differences on dominant phyla **(B)**, genera **(D)**, and species **(F)** between the wild and zoo groups were detected with the DESeq2 model.

At the phylum level, reads-based classification of the metagenomic sequences yielded an average alignment rate of 58.99%. The gut microbiota of Himalayan griffons was composed of seven dominant phyla, with the total relative abundances of more than 98%, namely *Fusobacteriota* (w group 41.65%, z group 38.45%), *Proteobacteria* (w group 22.91%, z group 25.42%), *Firmicutes_A* (w group 17.02%, z group 24.32%), *Bacteroidota* (w group 11.34%, z group 0.81%), *Firmicutes* (w group 2.48%, z group 4.73%), *Actinobacteriota* (w group 2.18%, z group 3.18%), and *Campylobacterota* (w group 1.16%, z group 1.22%) (Figure 2A). The comparison of microbial composition between the two groups at the phylum level was analyzed using DESeq2, with false-discovery rate (FDR) corrected *p* value ≤0.05 and log_2_ fold change ≥1. A total of seven phyla microbiome were more enriched in wild Himalayan griffons, while another five phyla were highly abundant in captive Himalayan griffons (Figure 2B).

From the genus perspective, an average of 41.14% of metagenomic sequences were not classified. The guts of Himalayan griffons were dominated by *Fusobacterium_A* (w group 39.05%, z group 37.93%), followed by *Escherichia* (w group 10.55%, z group 12.73%), *Plesiomonas* (w group 7.88%, z group 9.19%), *Clostridium_P* (w group 6.01%, z group 8.17%), *Clostridium* (w group 2.92%, z group 2.66%), and *Niameybacter* (w group 2.39%, z group 2.61%) (Figure 2C). In the case of differential genera, a total of 282 genera were identified as statistically different between the wild group and captive group (Figure 2D).

At the species level, the dominance of *Fusobacterium_A sp900015295* (w group 36.89%, z group 34.89%), *Escherichia coli* (w group 10.65%, z group 11.36%), *Plesiomonas shigelloides* (w group 7.90%, z group 9.24%), *Clostridium_P perfringens* (w group 6.04%, z group 8.30%), *UBA7332 sp002471225* (w group 5.72%, z group 0.00018%), and *Niameybacter stercoravium* (w group 2.19%, z group 2.43%) was identified (Figure 2E). A total of 2,214 species with statistical differences between the wild group and captive group were detected (Figure 2F).

### 3.3 Microbiome diversity analysis

**Figure 3:**
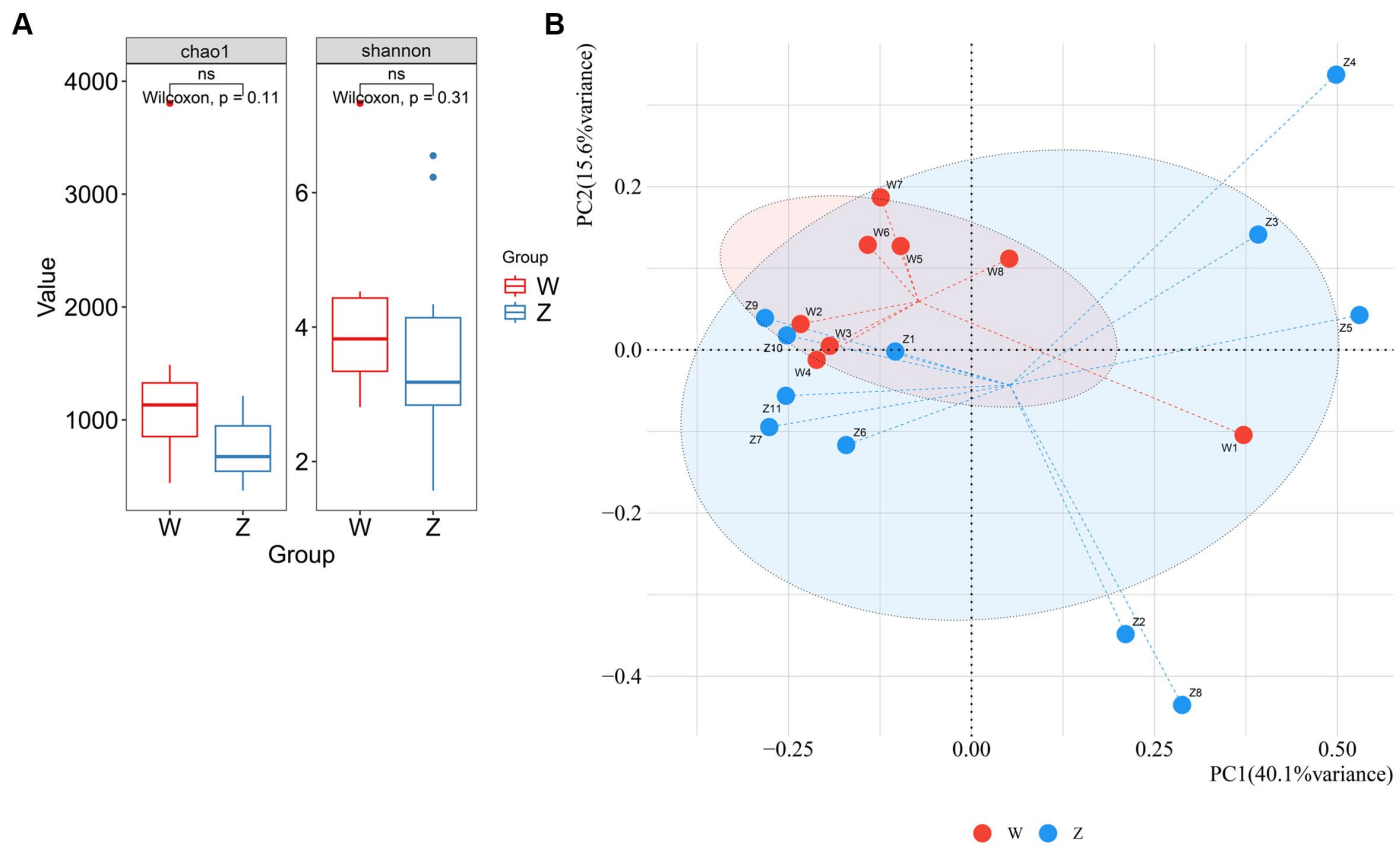
Comparison of alpha and beta diversity between the wild and zoo Himalayan griffons. **(A)** Boxplot showed the richness and Shannon index between the two groups. **(B)** Principal coordinates (PCoA) analysis with Bray-Curtis distance between the two groups.

The possible differences in alpha and beta diversity between the wild group and captive group were assessed based on metagenome sequences. Alpha diversity analysis using chao1 and shannon indices indicated that there was no significant difference between the two groups (Figure 3A). A PCoA plot based on the species-level relative abundances showed that axis 1 (PC1) explained 40.1% of the variability and axis 2 (PC2) explained 15.6% of the variability. The PCoA plot indicated the separation of samples from wild and captive Himalayan griffons (Figure 3B).

### 3.4 Functional profiling of Himalayan griffons microbiome

**Figure 4:**
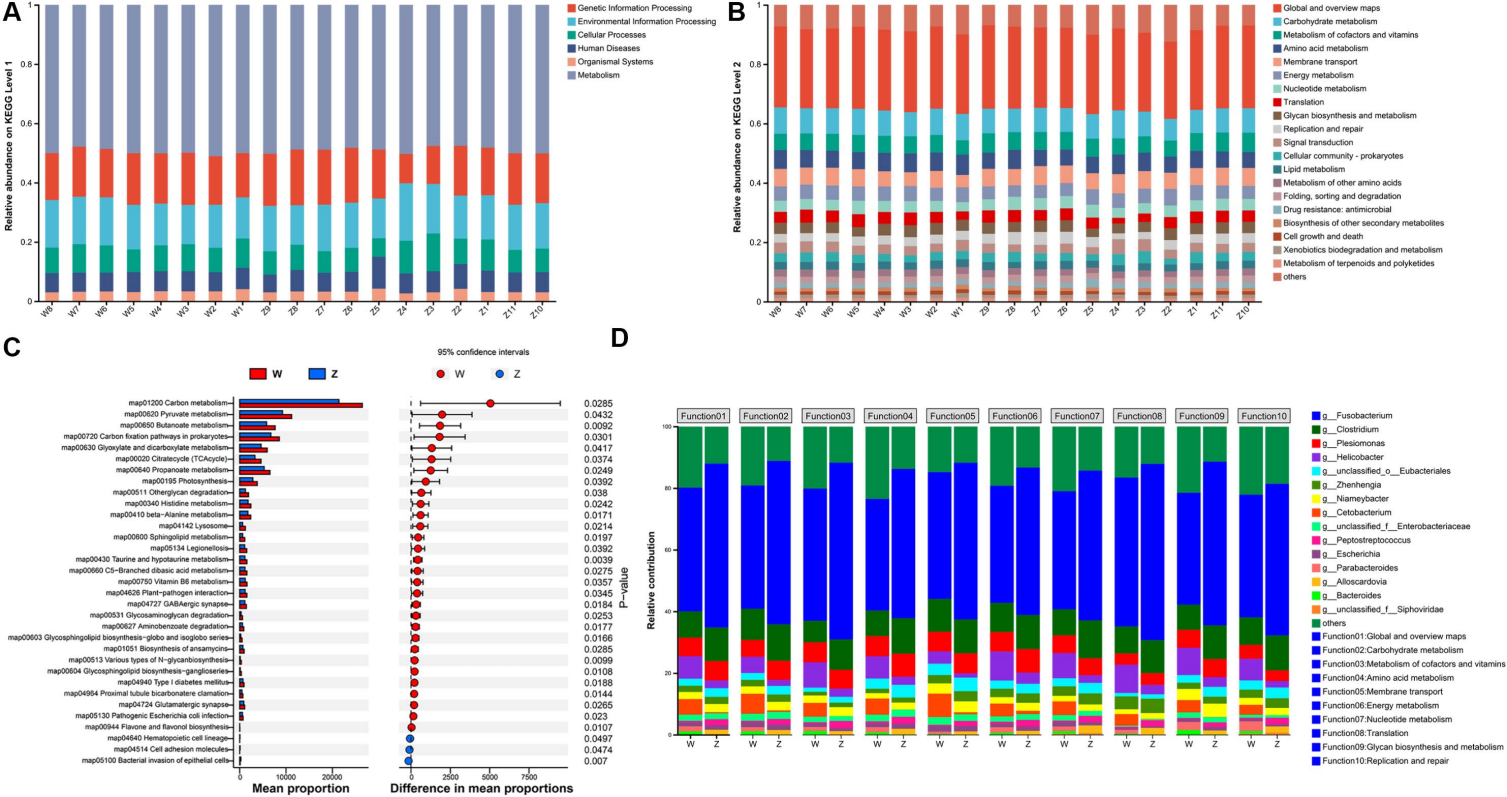
KEGG functional analysis of Himalayan griffons. Composition of KEGG level 1 **(A)** and level 2 **(B)** in the samples, and the significant differences in the relative abundance of KEGG pathway maps between the two groups **(C)**. **(D)** The main microbial genera contributed to KEGG functions at the level 2.

To further characterize the functional profiling of the Himalayan griffons gut microbiome, we predicted gene functions based on the KEGG database. A non-redundant reference gene catalog was build using the metagenome sequencing data and consisted of 4,869,648 unique genes. An average of 43.70% of the total mapped ORFs were assigned as KEGG pathway genes and a total of 459 metabolic pathways were identified. The metagenomic ORFs were classified into metabolism (47.59–51.08%), genetic information processing (9.91–18.76%), environmental information processing (13.25–19.39%), cellular processes (6.26–12.77%), human diseases (5.99–10.75%), and organismal systems (2.63–4.24%) (Figure 4A). At the level 2 (Figure 4B), the top five abundant categories under metabolism were carbohydrate metabolism, metabolism of cofactors and vitamins, amino acid metabolism, energy metabolism, and nucleotide metabolism. In the category of genetic information processing, the genes related to translation, and replication and repair were found in abundance. Membrane transport was the most abundant category under the environmental information processing. Difference analysis showed that there was a total of 30 differentially significant pathways enriched in the wild group, whereas another three pathways enriched in the captive group (Figure 4C). The predicted taxonomy involved in these abundant KEGG pathway was some of the gut microbial genera, such as *Fusobacterium*, *Clostridium*, *Plesiomonas*, *Helicobacter*, *Zhenhengia*, *Niameybacter*, *Cetobacterium*, *Peptostreptococcus*, *Escherichia*, and *Parabacteroides* (Figure 4D).

### 3.5 CAZymes analysis

**Figure 5:**
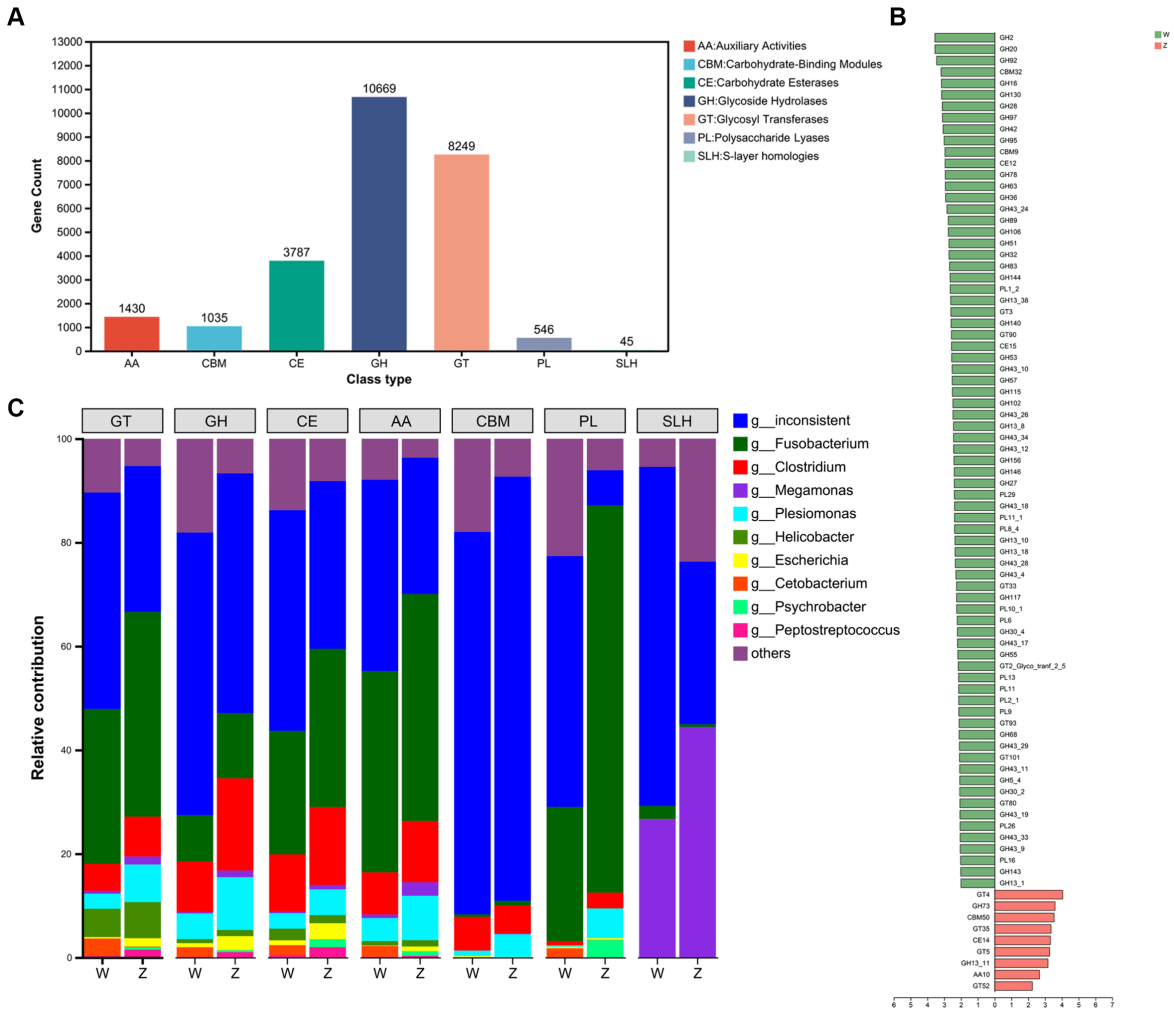
The carbohydrate-active enzymes (CAZymes) analysis of Himalayan griffons. The gene numbers of CAZymes types **(A)**, and Linear discriminant analysis (LDA) effect size (LEfSe) analysis of CAZymes between the wild and zoo groups **(B)**. **(C)** The main microbial genera contributed to different CAZymes types.

Then, the families of carbohydrate-active enzymes (CAZymes), which involved in forming, modifying, or hydrolyzing glycosidic bonds, in the gut microbiome of Himalayan griffons were analyzed using the CAZy database. A total of 25,761 CAZyme-encoding genes were obtained. These genes included 10,669 glycoside hydrolases (GHs), 8,249 glycosyltransferases (GTs), 3,787 carbohydrate esterases (CEs), 1,430 auxiliary activities (AA), 1,035 carbohydrate-binding modules (CBMs), 546 polysaccharide lyases (PLs), and 45 S-layer homologies (SLHs) (Figure 5A). The abundances of a total of 84 CAZymes were found to exhibit significant differences between the wild and captive groups (Figure 5B). Further, the CAZyme-encoding genes presented in major microbial genera were identified (Figure 5C). *Fusobacterium* (29.40%) and *Clostridium* (10.89%) together contributed to the largest number of CAZymes in our data set. Some members in GT families (e.g., GT9, GT41, GT2, and GT4) and CE families (e.g., CE9, CE1, and CE4) belonged in majority to *Fusobacterium*, and *Clostridium*.

### 3.6 Virulence factor genes and pathogenicity analysis

**Figure 6:**
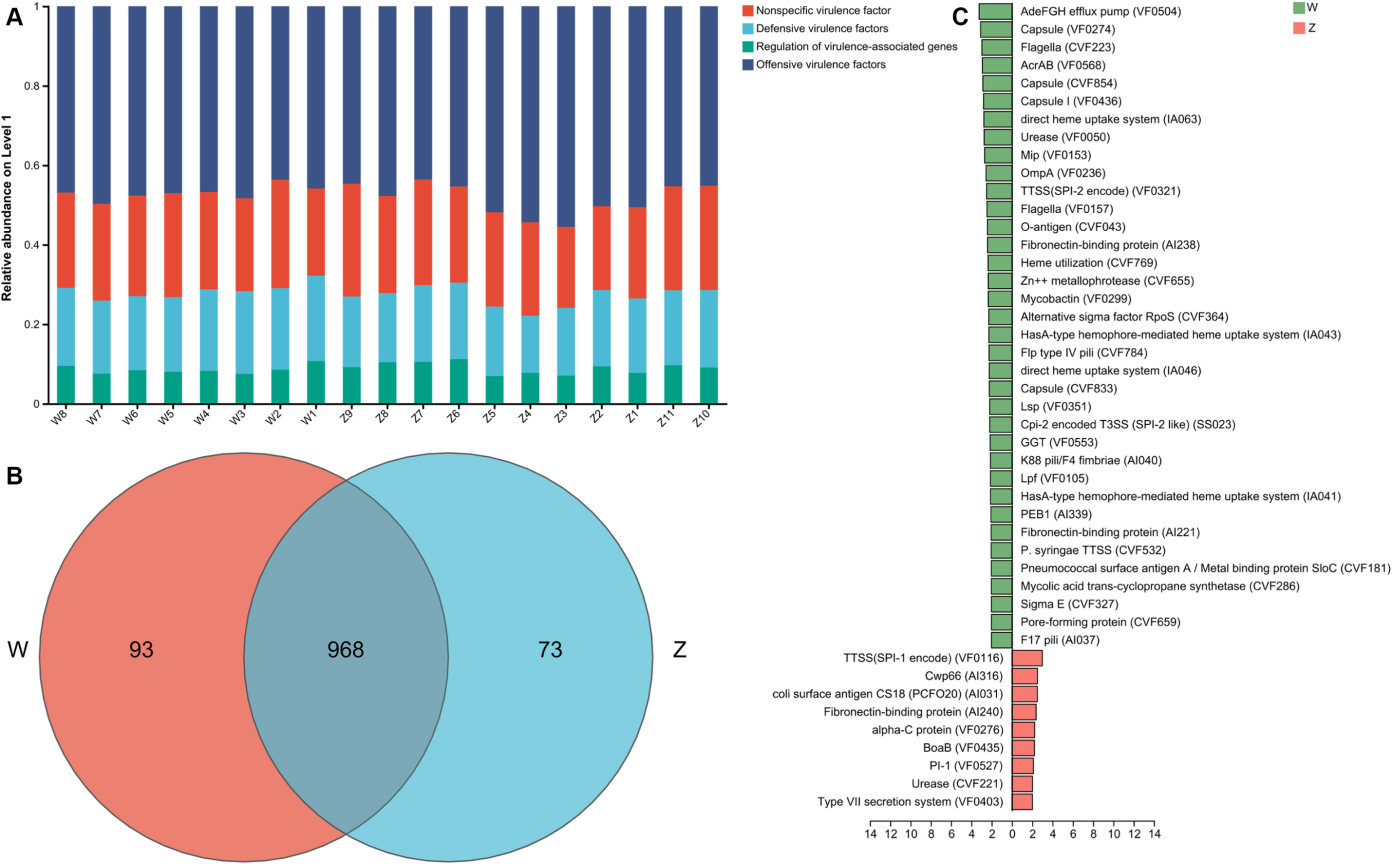
The virulence factor genes (VFGs) analysis of Himalayan griffons. **(A)** Composition of VFGs types in the samples. **(B)** The Venn diagram of the VFGs between the wild and zoo groups. **(C)** Linear discriminant analysis (LDA) effect size (LEfSe) analysis of VFGs between the wild and zoo groups.

It is crucial to identify the VFGs and the pathogenic bacteria of Himalayan griffons and explore their potential threat to public health. VFGs in the gut microbiome of Himalayan griffons were analyzed using virulence factor database, and offensive VFGs were found to be the dominant type among all samples (Figure 6A). The dominant subtypes belonged to offensive VFGs included adherence, secretion system, toxin, and invasion VFGs. The Venn diagram showed that two groups shared the vast majority of VFGs (Figure 6B). We also observed significant differences in VFGs based on the relative abundances in the gut microbiomes of wild and captive Himalayan griffons (Figure 6C).

**Figure 7:**
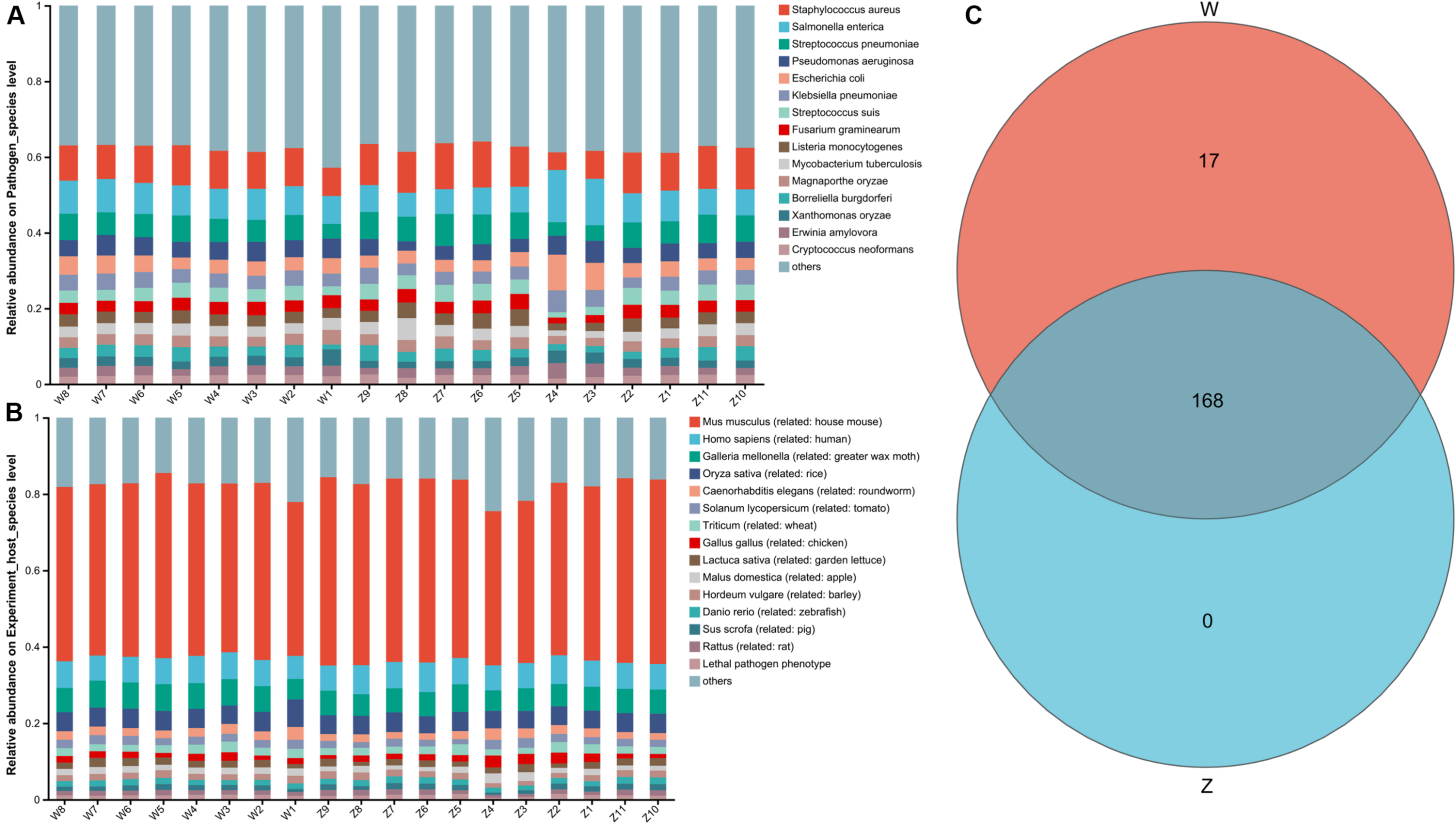
The pathogen host interactions (PHI) analysis of Himalayan griffons. Stacked bar graphs illustrated the top 15 abundant pathogen species **(A)** and the top 15 abundant host species **(B)**. **(C)** The Venn diagram of the pathogen species between the wild and zoo groups.

A total of 2,407 genes, 185 pathogen species, and 73 host species were annotated through the PHI (pathogen host interactions) database. The top 15 abundant pathogen species were showed in Figure 7A, where *Staphylococcus aureus*, *Salmonella enterica*, *Streptococcus pneumoniae*, *Pseudomonas aeruginosa*, and *Escherichia coli* were the main well-characterized bacterial pathogens. The top 15 abundant host species were showed in Figure 7B, with a list of animal host, such as house mouse, chicken, pig, and rat. The Venn diagram showed that two groups shared the vast majority of pathogen species, with no unique taxa in the zoo group (Figure 7C).

### 3.7 Reconstruction of MAGs from the metagenomes of Himalayan griffons

The metagenome-assembled genomes (MAGs) were reconstructed from all the 19 metagenomic sequencing data. A total of 130 non-redundant MAGs were obtained after removing redundant bins, with the completeness >50% and contamination rate < 10% (Supplementary Table S7). Among these, there were 40 MAGs with completeness of 50–70%, 44 MAGs with completeness of 70–90%, 44 MAGs with completeness (high-quality) > 90%, and two MAGs with completeness of 100%. Then, the 130 MAGs were classified using the genome taxonomy database toolkit (GTDB-tk). The results showed that all the MAGs were identified at the bacteria kingdom level, and all MAGs were classified at the order level at least (Table 1). All 130 MAGs were assigned to one kingdom, 12 phyla, 14 classes, and 30 orders. 129 MAGs (99.23%) were assigned to 44 families, and 115 MAGs (88.46%) were assigned to 60 genera. However, only 39 MAGs (30%) were identified as known microbial species, suggesting that majority of the MAGs (70%) were likely new species with no published genomes in the reference database.

**Table 1:** The taxonomic classification of MAGs in different levels.

Taxonomic level	Classified MAGs	Identified taxa	Unclassified MAGs
Kingdom	130	1	0
Phylum	130	12	0
Class	130	14	0
Order	130	30	0
Family	129	44	1
Genus	115	60	15
Species	39	23	91

**Figure 8:**
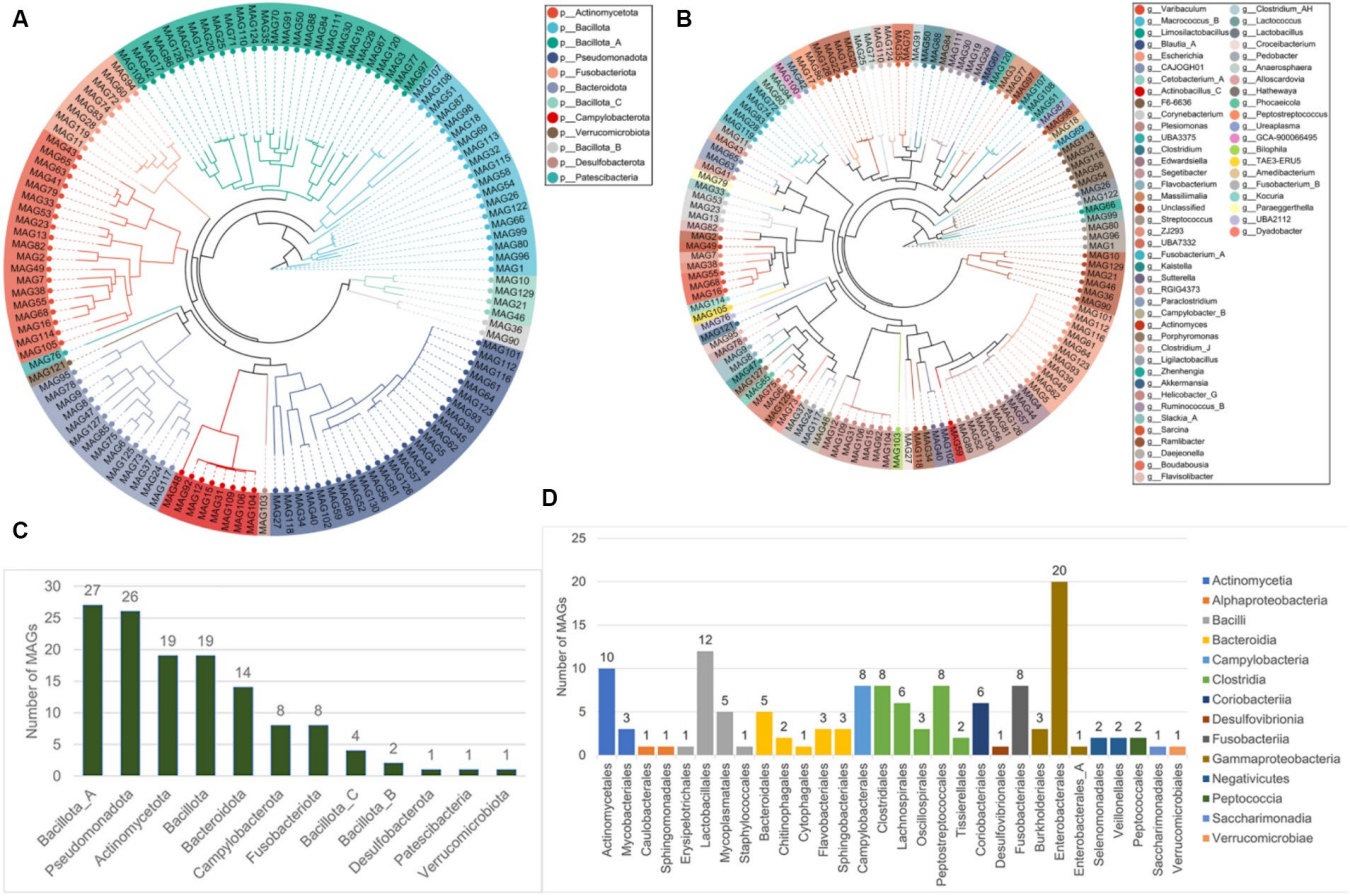
The taxonomic labels of MAGs. The phylogenetic tree of 130 MAGs at the phyla level **(A)** and the genera level **(B)**. **(C)** The number of MAGs at different 12 phyla, and at the different 14 classes and 30 orders **(D)**.

The phylogenetic tree of the 130 MAGs at the phyla and genera levels were constructed (Figures 8A,B). Most MAGs belonged to Bacillota (NCBI taxonomy: Firmicutes, *n* = 52), followed by Pseudomonadota (NCBI taxonomy: Proteobacteria, *n* = 26), Actinomycetota (NCBI taxonomy: Actinobacteria, *n* = 19), Bacteroidota (NCBI taxonomy: Bacteroides, *n* = 14) (Figure 8C). As shown in Figure 8D, the members in the phylum Bacillota contained the class Clostridia (*n* = 27) and Bacilli (*n* = 19). The members in Clostridia included the order Clostridiales (*n* = 8), Peptostreptococcales (*n* = 8), Lachnospirales (*n* = 6), Oscillospirales (*n* = 3), and Tissierellales (*n* = 2). The members in Bacilli included the order Lactobacillales (*n* = 12), Mycoplasmatales (*n* = 5), Erysipelotrichales (*n* = 1), and Staphylococcales (*n* = 1). Almost all the members in the phylum Pseudomonadota belonged to the class Gammaproteobacteria (*n* = 24), including the order Enterobacterales (*n* = 21) and Burkholderiales (*n* = 3). The members in the phylum Actinomycetota contained the class Actinomycetia (*n* = 13), and Coriobacteriia (*n* = 6). The members in Actinomycetia included the order Actinomycetales (*n* = 10) and Mycobacteriales (*n* = 3). All members of Bacteroidota belonged to the class Bacteroidia, including the order Bacteroidales (*n* = 5), Flavobacteriales (*n* = 3), Sphingobacteriales (*n* = 3), Chitinophagales (*n* = 2), and Cytophagales (*n* = 1).

## 4 Discussion

The pivotal role of griffons in ecosystem functioning and stability is extensively documented, owing to their scavenging feeding habits, with approximately 60–95% of carcasses being consumed by these avian species (65). In recent years, the decline of scavenger bird species has been linked to the weakening of the regulatory and cultural ecosystem services of these birds (66). Artificial food supplements have been one of the conservation tools used to support griffons during food scarcity, with the aim of protecting and recovering their populations (67). The artificially bred population in the zoo provides us with the feasibility to study the artificial feeding of Himalayan griffons in this region. However, the influence of artificial feeding on gut microbial communities of griffons remains unexplored. Our study provided the first comparative metagenomics of wild and captive Himalayan griffons to explore the effects of captivity conditions on the gut microbiota of this scavenger bird species. On the other hand, the gut metagenome data of many griffon species remain limited, so for the first time, we published metagenomic data for Himalayan griffons to fill in the gaps. Here, we presented a microbial gene catalog of the Himalayan griffons gut microbiome, which included 4,869,648 non-redundant genes, reconstructing a total of 130 microbial genomes. The comprehensive non-redundant gene catalog will provide a valuable reference resource for studying this bird species gut microbiome in the future. In addition, 70% of the MAGs represented strains with no genomes currently available in the reference database, potentially representing new species. Thus, the MAGs reported in this paper are another valuable resource that will significantly increase the number of uncultured microbes’ genomes, and provide clues for a comprehensive understanding of the complex gut environments.

Species composition analysis revealed the unique gut microbial structures of Himalayan griffons in this study, such as the high-abundance of Fusobacteriota, which is significantly different from the gut microbiota commonly found in other birds (14, 15, 68). Consistent with other previous studies on the gut microbiome of scavengers (42, 69), Fusobacteriota and Proteobacteria were the two most dominant phyla. The unique characteristics of the Himalayan griffons gut environment may be closely related to their scavenging dietary characteristics. The food sources of Himalayan griffons in the wild can be complex, including the carcasses of both wild animals and domestic animals. Some of the microbes that break down the carcass are able to excrete toxic metabolites, which in turn make the carcass a dangerous food source for most carnivorous and omnivorous animals. However, griffons are able to eat carrion without any apparent ill effects. Most bacteria that enter a bird host are transient or short-lived, while some bacteria can acquire a niche that allowing colonization, which may explain how the bird gut microbiome is shaped and structured (16). The high frequency of Fusobacteriota and Proteobacteria was detected in the gut of Himalayan griffons, indicating that members of these phyla could survive in the acidic gastric passage before colonizing the hindgut, and finally retained by the host. We hypothesized that these gut microbes might be derived from carcasses. To confirm this hypothesis, the challenge for the future lies in obtaining more high-quality genomic information on various foodborne gut bacteria to support the comparative genomic analysis and studies of the origins of some gut microbial members.

In contrast to the wild Himalayan griffons, the captive populations received a relatively stable and clean diet and a fixed living environment. A total of 12 phyla, 282 genera, and 2,214 species with statistical differences were detected between the wild group and captive group. These differences in the bacterial compositions suggested that various survival environments and diets had different constructing effects on the commensal microbiota of the host. In addition to diet, other aspects of captivity, including limited habitat space, contact with humans, and medications (such as antibiotics) are known to affect animal gut microbiome (32). The gut microbiota that did not change in the two comparison groups might be due to the fact that these captive individuals came from the wild and had already formed their own stable gut microbiome before entering the zoo. These fixed gut microbiome also suggested that artificial-feeding or re-release of captive individuals is feasible for Himalayan griffon species.

Contrary to some previous studies, but consistent with some others, we did not observe lower gut microbiota diversity in captive griffons than in wild populations. Such inconsistencies raise questions about the widely held view that wild populations have greater alpha diversity. For instance, the alpha diversity was significantly higher in wild populations of certain birds, including the oriental white stork (*Ciconia boyciana*) (28) and the Chinese monal (*Lophophorus lhuysii*) (70), when compared to their counterparts in captivity. Greatest alpha diversity was also found in some other wild mammals, such as alpine musk deer (*Moschus chrysogaster*) (71), bharals (*Pseudois nayaur*) (72), snub-nosed monkeys (*Rhinopithecus roxellana*) (73), deer mice (*Peromyscus maniculatus*) (74), the Przewalski’s Horse (*Equus ferus przewalskii*), and the Asian wild ass (*Equus hemionus*) (75), while the captive ones had the least alpha diversity. However, for some species, no difference in alpha diversity was found between wild and domestic populations, or even higher alpha diversity was found in domestic populations. For example, alpha diversity in raptors of seven different species (belonged to orders *Strigiformes*, *Accipitriformes*, and *Falconiformes*) was not affected by captivity (29). Similarly, the largest alpha diversity was detected in the captive red-crowned cranes (*Grus japonensis*), while wild cranes had the least alpha diversity (76). The same phenomenon was also found in other mammals, such as the ring-tailed lemur (*Lemur catta*) (77), the black rhinoceros (*Diceros bicornis*) (78), and the rhesus macaques (*Macaca mulatta*) (79). These results increased more and more evidence that alpha diversity should not be used alone to infer wild or captive conditions for griffons species. On the other hand, it was reported that griffons that fed on carrion were thought to protect them from extraneous pathogens and bacterial toxins by having an extremely low stomach pH (38, 41). Bacteria from food or the environment were strong filtered by the acidic gastrointestinal tract of griffons, which might explain why alpha diversity did not differ significantly between the two groups.

Compared to typical avian microbiota profiles, a lot of microbial members in Himalayan griffons detected in this study were documented as the cause of severe food poisoning in both humans and other animals. For example, a total of 107 strains of *Clostridium perfringens* were isolated from the three griffon species (69). This raises the question of whether the selective retention of these bacteria by griffons has any physiological benefits. Microbial functional analysis based on KEGG databases showed enrichment of metabolic functions related to carbohydrate, amino acid, and other substances in the gut microbiota, indicating higher levels of energy requirements. The wild group showed more diverse metabolic differences than the zoo group, indicating a more abundant food resources in the wild group. In addition, wild Himalayan griffons had a lot of space to move around and had high-energy requirements for flight. Captive populations, on the other hand, had little space and rarely fly. Abundant CAZymes were found to be in Himalayan griffons guts, especially the presence of GHs and GTs, which are the key enzyme families for carbohydrates degradation (80), which might further indicate that more active gut microbiome metabolism of carbohydrates. Interestingly, we found that *Fusobacterium* and *Clostridium* together contributed to the largest number of CAZymes in our data set. This observation provided indirect evidence suggesting that these pathogens may exert certain physiological effects in support of the host. Considering that Himalayan griffons are carnivorous birds, the source and function of CAZymes need to be further studied in conjunction with their dietary composition. Egyptian vultures (*Neophron percnopterus*) were reported to have unusual coprophagic tendencies, and contained insects in their diets. This might be a source of fiber intake by Himalayan griffons, both from plant (e.g., prey digestive tracts) and animal (e.g., skin, bone, chitin, and connective tissue) sources (81). However, we currently lack data on the food composition of Himalayan griffons, which could be studied in the future using eDNA technology.

Gut microbiomes can inhibit the invasion of pathogens through direct and indirect interactions, and thus forming the first line of defense against invasion (82, 83). Therefore, we speculate that Himalayan griffons may rely on the symbiotic indigenous gut microbes to outcompete other bacterial groups derived from the scavenging diets, thus providing another beneficial effects of gut microbiota on the Himalayan griffons. Furthermore, offensive VFGs were found to be the dominant type in all samples in our study, while the defensive VFGs accounted for a relatively small proportion. Offensive VFGs were known to have aggressive functions, such as directly damaging the host, while the defensive VFGs could enhance the immune system endurance (84). The host bacteria with offensive VFGs in Himalayan griffons had a stronger attack ability. Scavenging birds were reported to contain antibodies against toxins such as botulinum (85), and we hypothesized that Himalayan griffons had unusually tolerance to toxins released by offensive pathogens. The genomic analyses related to Himalayan griffons showed that they had strong immune systems with a wide range of positive selection on immune genes, which may be another reason why Himalayan griffons host could co-exist with so many pathogens (38). Pathogens carrying large amounts of VFGs that are widespread in animal intestines would pose a threat to public health. Through the analysis of PHI database, we also found some key pathogens and corresponding hosts in the gut microbial communities of Himalayan griffons, which pointed out the direction for monitoring the transmission of pathogens of associated with this bird species in the future (86).

## 5 Conclusion

This is the first study to characterize the gut microbiome of Himalayan griffons under different conditions (wild vs. captive) using metagenomic sequencing analysis. The present study provided a first inventory of the microbial genes and metagenome-assembled genomes related to Himalayan griffons. Comparing analysis identified some variations of gut microbiota taxonomic and functional features between captive and wild populations. According to our results, a total of seven bacterial phyla were more prevalent in wild Himalayan griffons, while another five phyla were more common in captive Himalayan griffons. A total of 282 genera and 2,214 species with statistical differences were detected between the wild group and the captive group. Additionally, the gut microbiota of the two groups exhibited functional differences, with 33 KEGG pathways and 84 CAZyme families being significantly altered between wild and captive individuals. The genetic factors were the same, therefore it was hypothesized that the differences were mainly associated with the environment and diet. These findings were of great significance for the reintroduction of captive Himalayan griffons, and improving conservation and management strategies for this near threatened species. The results will also help to inform future studies on the health protective effects of the microbiota on scavengers.

## Data availability statement

The raw sequence data reported in this paper have been deposited in the Genome Sequence Archive in National Genomics Data Center, China National Center for Bioinformation/Beijing Institute of Genomics, Chinese Academy of Sciences (GSA: CRA012991) and are publicly accessible at https://ngdc.cncb.ac.cn/gsa.

## Ethics statement

The research protocol was reviewed and approved by the Ethical Committee of Qinghai University. This study did not involve capture or any direct manipulation or disturbance of Himalayan griffons. The studies were conducted in accordance with the local legislation and institutional requirements. Written informed consent was obtained from the owners for the participation of their animals in this study.

## Author contributions

YW: Conceptualization, Data curation, Formal Analysis, Investigation, Methodology, Resources, Software, Validation, Visualization, Writing – original draft. JZ: Conceptualization, Data curation, Formal Analysis, Investigation, Methodology, Resources, Software, Validation, Visualization, Writing – original draft. BT: Conceptualization, Data curation, Formal Analysis, Investigation, Methodology, Resources, Software, Validation, Visualization, Writing – original draft. YD: Conceptualization, Data curation, Formal Analysis, Investigation, Methodology, Resources, Software, Validation, Visualization, Writing – original draft. SS: Conceptualization, Data curation, Formal Analysis, Investigation, Methodology, Resources, Software, Validation, Visualization, Writing – original draft. SH: Conceptualization, Data curation, Formal Analysis, Investigation, Methodology, Resources, Software, Validation, Visualization, Writing – original draft. WZ: Conceptualization, Data curation, Formal Analysis, Investigation, Methodology, Resources, Software, Validation, Visualization, Writing – original draft. ZL: Conceptualization, Data curation, Formal Analysis, Resources, Validation, Visualization, Writing – review & editing. QJ: Conceptualization, Data curation, Formal Analysis, Resources, Validation, Visualization, Writing – original draft, Writing – review & editing. WW: Conceptualization, Data curation, Formal Analysis, Funding acquisition, Project administration, Resources, Supervision, Validation, Visualization, Writing – original draft, Writing – review & editing.

## Footnotes

^1^https://github.com/biobakery/kneaddata

^2^http://www.cazy.org/

^3^http://www.mgc.ac.cn/VFs/

^4^http://www.phi-base.org/index.jsp
