# Supplementary material for: Metagenomic comparison of gut communities between wild and captive Himalayan griffons
Source: Front Vet Sci. 2024 May 9;11:1403932. doi: 10.3389/fvets.2024.1403932 (PMC11112026; doi:10.3389/fvets.2024.1403932)
Supplement: Supplementary file 1 [file Table_1.DOCX]

Supplementary Material

Metagenomic comparison of gut communities between wild and captive Himalayan griffons

You Wang^1,2,†^, Jundie Zhai^1,2,†^, Boyu Tang^1,2^, Yonggang Dong^1,2^, Shengzhen Sun^3^, Mengjun Wei^3^, Shunfu He^4^, Wenxin Zhao^4^, Zhuoma Lancuo^5^, Alexey Druzyaka^6^, Kirill Sharshov^7^, Qiangqiang Jia^1,*^, Wen Wang^1,*^

*** Correspondence:** Qiangqiang Jia: jia1108029@163.com; Wen Wang: [007cell@163.com](mailto:jia1108029@163.com)

# Supplementary Tables

**Supplementary S1.** Summary of the sequencing data for each sample.

| Samples | Raw reads | Raw base | Clean reads | Clean base |
| --- | --- | --- | --- | --- |
| W1 | 23,239,411 | 6,971,823,300 | 18,460,526 | 5,495,156,819 |
| W2 | 30,627,900 | 9,188,370,000 | 29,745,735 | 8,829,512,169 |
| W3 | 28,324,893 | 8,497,467,900 | 27,721,992 | 8,219,852,331 |
| W4 | 26,453,550 | 7,936,065,000 | 25,969,636 | 7,707,111,028 |
| W5 | 27,309,293 | 8,192,787,900 | 26,556,076 | 7,880,374,049 |
| W6 | 10,877,955 | 3,222,619,109 | 6,110,595 | 1,833,178,500 |
| W7 | 23,787,083 | 7,136,124,900 | 18,005,223 | 5,335,394,751 |
| W8 | 26,914,933 | 8,074,479,900 | 26,303,543 | 7,785,576,569 |
| Z1 | 30,242,672 | 9,072,801,600 | 17,400,402 | 5,158,519,441 |
| Z2 | 38,781,064 | 11,634,319,200 | 6,317,036 | 1,863,972,494 |
| Z3 | 29,331,702 | 8,799,510,600 | 17,654,582 | 5,225,195,615 |
| Z4 | 43,824,952 | 13,147,485,600 | 12,528,282 | 3,689,859,110 |
| Z5 | 22,218,041 | 6,665,412,300 | 16,287,726 | 4,782,235,294 |
| Z6 | 22,326,304 | 6,697,891,200 | 21,940,544 | 6,481,611,508 |
| Z7 | 19,006,112 | 5,701,833,600 | 18,547,660 | 5,475,576,675 |
| Z8 | 17,811,978 | 5,343,593,400 | 17,411,797 | 5,132,915,416 |
| Z9 | 17,604,634 | 5,281,390,200 | 17,261,060 | 5,070,386,385 |
| Z10 | 17,948,958 | 5,384,687,400 | 17,625,673 | 5,194,199,204 |
| Z11 | 18,626,599 | 5,587,979,700 | 18,361,477 | 5,422,110,638 |

**Supplementary S2.** Summary of assembly statistics for each sample.

| Samples | Total assembly length (bp) | Number of contigs | Number of contigs > 1 kb | Largest contig (bp) | GC content (%) | N50 |
| --- | --- | --- | --- | --- | --- | --- |
| W1 | 280,991,186 | 241,149 | 59,143 | 366,531 | 49.69 | 1,217 |
| W2 | 102,737,875 | 52,792 | 19,598 | 667,245 | 35.07 | 4,088 |
| W3 | 105,179,463 | 50,907 | 19,625 | 627,441 | 36.52 | 4,961 |
| W4 | 115,080,555 | 51,133 | 22,824 | 582,069 | 35.59 | 4,962 |
| W5 | 105,352,846 | 71,813 | 18,046 | 447,644 | 36.05 | 2,219 |
| W6 | 53,934,575 | 36,753 | 12,289 | 601,901 | 37.35 | 1,818 |
| W7 | 78,930,746 | 52,547 | 17,045 | 481,126 | 37.40 | 2,067 |
| W8 | 153,365,308 | 75,111 | 27,069 | 532,998 | 38.22 | 5,216 |
| Z1 | 79,038,134 | 54,334 | 17,146 | 238,422 | 35.64 | 1,980 |
| Z2 | 64,707,077 | 50,770 | 14,524 | 332,496 | 36.01 | 1,432 |
| Z3 | 104,336,352 | 79,063 | 20,244 | 277,596 | 39.29 | 1,619 |
| Z4 | 80,435,221 | 64,003 | 17,451 | 100,846 | 47.48 | 1,534 |
| Z5 | 121,043,756 | 169,423 | 9,227 | 150,500 | 49.74 | 647 |
| Z6 | 72,131,398 | 27,277 | 12,307 | 457,788 | 31.95 | 7,701 |
| Z7 | 64,835,233 | 31,159 | 14,206 | 652,956 | 33.23 | 3,822 |
| Z8 | 114,094,877 | 60,070 | 22,408 | 750,505 | 40.48 | 3,922 |
| Z9 | 37,116,903 | 18,065 | 6,280 | 229,313 | 37.24 | 5,954 |
| Z10 | 69,636,458 | 40,104 | 13,433 | 401,743 | 35.17 | 3,156 |
| Z11 | 83,144,797 | 48,522 | 17,788 | 521,046 | 34.88 | 2,853 |

**Supplementary S3.** Summary of predicted unigenes data in each sample.

| Samples | Total length of unigenes (bp) | Number of unigenes | Average length of unigenes (bp) | Minimun length (bp) | Maximum length (bp) |
| --- | --- | --- | --- | --- | --- |
| W1 | 401,424,195 | 976,921 | 410.91 | 60 | 24,072 |
| W2 | 108,843,105 | 199,276 | 546.19 | 60 | 15,678 |
| W3 | 109,942,167 | 205,050 | 536.17 | 60 | 15,417 |
| W4 | 115,881,984 | 205,416 | 564.13 | 60 | 15,789 |
| W5 | 144,558,795 | 333,712 | 433.18 | 60 | 15,831 |
| W6 | 60,108,240 | 121,011 | 496.72 | 60 | 9,762 |
| W7 | 87,625,485 | 176,651 | 496.04 | 60 | 11,688 |
| W8 | 166,216,584 | 314,570 | 528.39 | 60 | 11,757 |
| Z1 | 90,500,886 | 192,161 | 470.96 | 60 | 11,352 |
| Z2 | 75,899,634 | 180,202 | 421.19 | 60 | 11,604 |
| Z3 | 126,513,972 | 314,805 | 401.88 | 60 | 14,652 |
| Z4 | 92,021,601 | 245,817 | 374.35 | 60 | 21,669 |
| Z5 | 124,604,832 | 538,882 | 231.23 | 60 | 12,693 |
| Z6 | 73,694,670 | 128,489 | 573.55 | 60 | 10,329 |
| Z7 | 65,172,099 | 115,042 | 566.51 | 60 | 11,376 |
| Z8 | 123,322,935 | 228,962 | 538.62 | 60 | 16,206 |
| Z9 | 39,646,989 | 73,842 | 536.92 | 60 | 8,892 |
| Z10 | 76,094,880 | 145,260 | 523.85 | 60 | 11,589 |
| Z11 | 90,167,634 | 173,579 | 519.46 | 60 | 11,904 |

**Supplementary S4.** The number of reads assigned to different phylum in each sample.

| Phylum | W1 | W2 | W3 | W4 | W5 | W6 | W7 | W8 | Z1 | Z2 | Z3 | Z4 | Z5 | Z6 | Z7 | Z8 | Z9 | Z10 | Z11 |
| --- | --- | --- | --- | --- | --- | --- | --- | --- | --- | --- | --- | --- | --- | --- | --- | --- | --- | --- | --- |
| Proteobacteria | 4040822 | 1454430 | 1923242 | 1565318 | 3269839 | 2240998 | 4885472 | 4247669 | 1931549 | 415452 | 8833113 | 6590159 | 1648592 | 290739 | 343907 | 390352 | 1174589 | 1194060 | 771095 |
| Firmicutes_A | 2057188 | 2299833 | 2461327 | 2786328 | 3196625 | 1258438 | 1414911 | 3830528 | 1938916 | 1366208 | 2795042 | 385854 | 724591 | 7070419 | 3275431 | 1970438 | 539176 | 1572754 | 1952110 |
| Actinobacteriota | 1118378 | 5210 | 15415 | 10377 | 136617 | 3729 | 8055 | 790240 | 65013 | 153159 | 136214 | 310452 | 124943 | 5261 | 125805 | 950526 | 44704 | 116690 | 133820 |
| Bacteroidota | 993172 | 5044871 | 2753573 | 2864458 | 40505 | 154698 | 90830 | 2720266 | 46357 | 59220 | 90346 | 33729 | 71451 | 20484 | 37459 | 47207 | 22627 | 109824 | 60146 |
| Fusobacteriota | 756142 | 10231763 | 6549120 | 6941189 | 8248249 | 4122045 | 6435835 | 5577566 | 4130176 | 261483 | 811470 | 14505 | 60288 | 6162294 | 6967292 | 1459989 | 11313821 | 8721882 | 8346959 |
| Acidobacteriota | 64936 | 69 | 434 | 240 | 153 | 0 | 71 | 184 | 396 | 840 | 704 | 2399 | 973 | 0 | 692 | 267 | 69 | 94 | 39 |
| Verrucomicrobiota | 48442 | 883 | 879 | 660 | 586 | 36728 | 1357 | 12435 | 1153 | 2135 | 418 | 712 | 1998 | 0 | 359 | 334 | 48 | 32 | 60 |
| Firmicutes | 78781 | 368164 | 794075 | 693341 | 308259 | 93870 | 182713 | 524763 | 439974 | 145198 | 244906 | 19066 | 212558 | 551401 | 267649 | 1110223 | 144439 | 407817 | 455232 |
| Planctomycetota | 24824 | 213 | 578 | 271 | 267 | 64 | 158 | 925 | 1061 | 788 | 1404 | 1514 | 2278 | 0 | 985 | 1855 | 66 | 289 | 138 |
| Myxococcota | 18081 | 0 | 52 | 46 | 116 | 0 | 40 | 95 | 496 | 389 | 790 | 870 | 1163 | 0 | 1967 | 871 | 821 | 178 | 1082 |
| Patescibacteria | 10589 | 2481 | 3149 | 2151 | 4859 | 659 | 848 | 2286 | 1910 | 2252 | 712 | 562 | 794 | 452 | 652 | 781 | 326 | 220 | 501 |
| Chloroflexota | 9403 | 925 | 1343 | 987 | 2191 | 490 | 1112 | 1018 | 1900 | 3384 | 1052 | 1586 | 1594 | 775 | 1165 | 2231 | 1413 | 1232 | 1196 |
| Campylobacterota | 11427 | 24123 | 817466 | 423729 | 82140 | 13409 | 32125 | 6024 | 382689 | 140039 | 51630 | 160 | 97743 | 431 | 648 | 1885 | 1361 | 9019 | 52896 |
| Gemmatimonadota | 7605 | 0 | 68 | 0 | 38 | 0 | 0 | 0 | 254 | 319 | 347 | 1421 | 404 | 0 | 1076 | 146 | 189 | 164 | 69 |
| Methylomirabilota | 5432 | 0 | 0 | 0 | 0 | 0 | 0 | 0 | 0 | 0 | 0 | 72 | 46 | 0 | 0 | 0 | 0 | 0 | 0 |
| Cyanobacteria | 3600 | 4306 | 3786 | 3204 | 5673 | 1123 | 1518 | 2370 | 4828 | 5519 | 14429 | 27820 | 20402 | 895 | 891 | 1317 | 1047 | 1039 | 1196 |
| Desulfobacterota_B | 3424 | 0 | 542 | 278 | 57 | 0 | 0 | 0 | 229 | 118 | 0 | 95 | 0 | 0 | 0 | 0 | 0 | 0 | 0 |
| Nitrospirota | 2988 | 355 | 287 | 180 | 159 | 114 | 83 | 166 | 203 | 107 | 121 | 263 | 145 | 0 | 78 | 0 | 0 | 0 | 0 |
| Armatimonadota | 1410 | 7 | 41 | 26 | 29 | 0 | 0 | 33 | 19 | 0 | 46 | 54 | 85 | 0 | 0 | 0 | 0 | 0 | 0 |
| Desulfobacterota | 1010 | 327 | 373 | 303 | 220 | 80 | 127 | 264 | 462 | 176 | 180 | 106 | 384 | 0 | 49 | 73 | 0 | 39 | 0 |
| Firmicutes_C | 2560 | 4479 | 16167 | 10724 | 13119 | 9450 | 29986 | 18969 | 58316 | 24318 | 48901 | 2234 | 17313 | 1265 | 2269 | 19360 | 1521 | 2033 | 2708 |
| Bdellovibrionota | 924 | 359 | 784 | 426 | 218 | 122 | 141 | 216 | 387 | 504 | 208 | 82 | 400 | 578 | 97 | 161 | 148 | 1742 | 1575 |
| Myxococcota_A | 728 | 0 | 0 | 0 | 0 | 0 | 0 | 0 | 0 | 0 | 0 | 36 | 87 | 0 | 0 | 0 | 0 | 0 | 0 |
| Spirochaetota | 653 | 1265 | 1563 | 1155 | 1854 | 454 | 575 | 2240 | 1256 | 362 | 19602 | 2801 | 286023 | 283 | 212 | 1243 | 193 | 180 | 258 |
| Firmicutes_B | 628 | 526 | 1213 | 834 | 375 | 254 | 580 | 7329 | 1524 | 393 | 527 | 40 | 235 | 903 | 223 | 3759 | 142 | 198 | 509 |
| Deinococcota | 293 | 0 | 7 | 0 | 44 | 0 | 0 | 0 | 1605 | 8899 | 27846 | 1224 | 1241 | 0 | 4093 | 1457 | 101 | 54 | 278 |
| Eisenbacteria | 320 | 0 | 0 | 0 | 0 | 0 | 0 | 0 | 0 | 0 | 0 | 0 | 0 | 0 | 0 | 0 | 0 | 0 | 0 |
| Desulfobacterota_I | 248 | 103 | 89 | 66 | 178 | 892 | 357 | 835930 | 171 | 127 | 124 | 111 | 191 | 0 | 60 | 0 | 57 | 35 | 1821 |
| Dormibacterota | 279 | 0 | 0 | 0 | 0 | 0 | 0 | 0 | 0 | 0 | 0 | 0 | 59 | 0 | 0 | 0 | 0 | 0 | 0 |
| Chlamydiota | 336 | 315 | 364 | 270 | 135 | 104 | 222 | 133 | 14660 | 46005 | 88573 | 2610 | 38882 | 0 | 164 | 5366 | 0 | 0 | 0 |
| Elusimicrobiota | 190 | 55 | 122 | 75 | 72 | 0 | 0 | 71 | 188 | 28 | 42 | 0 | 124 | 0 | 0 | 0 | 0 | 0 | 0 |
| Eremiobacterota | 173 | 0 | 0 | 0 | 0 | 0 | 0 | 0 | 0 | 0 | 0 | 0 | 0 | 0 | 0 | 0 | 0 | 0 | 0 |
| Fibrobacterota | 159 | 0 | 113 | 94 | 333 | 0 | 35 | 1177 | 44 | 0 | 0 | 0 | 53 | 0 | 0 | 0 | 0 | 0 | 0 |
| Omnitrophota | 111 | 42 | 40 | 44 | 0 | 0 | 0 | 46 | 37 | 0 | 0 | 0 | 65 | 0 | 0 | 0 | 0 | 0 | 0 |
| Marinisomatota | 122 | 371 | 140 | 159 | 103 | 0 | 48 | 111 | 231 | 0 | 39 | 0 | 504 | 0 | 0 | 226 | 0 | 0 | 0 |
| Hydrogenedentota | 76 | 0 | 0 | 0 | 0 | 0 | 0 | 0 | 0 | 0 | 0 | 0 | 0 | 0 | 0 | 0 | 0 | 0 | 0 |
| Thermotogota | 261 | 368 | 411 | 232 | 158 | 643 | 2821 | 236 | 4474 | 0 | 0 | 0 | 33 | 9896 | 0 | 31774 | 1580 | 563 | 3023 |
| Krumholzibacteriota | 68 | 0 | 0 | 0 | 0 | 0 | 0 | 0 | 0 | 0 | 0 | 0 | 0 | 0 | 0 | 0 | 0 | 0 | 0 |
| Synergistota | 65 | 0 | 159 | 96 | 82 | 56 | 92 | 96 | 55 | 0 | 0 | 0 | 15145 | 0 | 0 | 235 | 0 | 150 | 202 |
| Firmicutes_F | 84 | 237 | 243 | 241 | 221 | 79 | 79 | 191 | 222 | 58 | 104 | 0 | 59 | 84 | 93 | 351 | 0 | 42 | 0 |
| Nitrospinota | 48 | 0 | 0 | 0 | 0 | 0 | 0 | 48 | 0 | 0 | 0 | 0 | 0 | 0 | 0 | 0 | 0 | 0 | 0 |
| Latescibacterota | 51 | 0 | 0 | 0 | 0 | 0 | 0 | 0 | 0 | 0 | 0 | 0 | 0 | 0 | 0 | 0 | 0 | 0 | 0 |
| Firmicutes_G | 44 | 1137 | 258 | 302 | 47 | 0 | 0 | 57 | 77 | 0 | 0 | 0 | 56 | 0 | 0 | 0 | 0 | 0 | 0 |
| KSB1 | 37 | 0 | 0 | 0 | 0 | 0 | 0 | 0 | 0 | 0 | 0 | 0 | 0 | 0 | 0 | 0 | 0 | 0 | 0 |
| SAR324 | 55 | 0 | 95 | 0 | 0 | 0 | 0 | 32 | 74 | 0 | 125 | 55 | 195 | 0 | 0 | 9 | 0 | 0 | 0 |
| CSP1-3 | 70 | 0 | 0 | 0 | 0 | 0 | 0 | 0 | 0 | 0 | 0 | 0 | 0 | 0 | 0 | 0 | 0 | 0 | 0 |
| Methanobacteriota | 20611 | 12199 | 15780 | 18190 | 98670 | 1214 | 420 | 29772 | 347 | 49 | 48 | 0 | 1018 | 585 | 0 | 331 | 0 | 0 | 121 |
| Thermoproteota | 5314 | 330 | 440 | 340 | 289 | 58 | 139 | 217 | 887 | 660 | 707 | 1183 | 1782 | 87 | 373 | 3942 | 0 | 161 | 52 |
| Halobacteriota | 533 | 174 | 121 | 91 | 92 | 0 | 0 | 157 | 327 | 73 | 80 | 116 | 262 | 0 | 0 | 0 | 0 | 0 | 0 |
| Nanoarchaeota | 152 | 455 | 292 | 403 | 222 | 123 | 197 | 291 | 142 | 0 | 42 | 0 | 127 | 165 | 0 | 179 | 164 | 72 | 226 |
| Asgardarchaeota | 156 | 310 | 283 | 265 | 277 | 170 | 180 | 334 | 334 | 118 | 49 | 0 | 213 | 48 | 43 | 83 | 79 | 56 | 68 |
| Thermoplasmatota | 146 | 118 | 270 | 289 | 107 | 0 | 159 | 234 | 229 | 224 | 0 | 0 | 119 | 0 | 0 | 0 | 0 | 0 | 0 |
| JACRDZ01 | 0 | 175 | 146 | 159 | 154 | 93 | 117 | 143 | 56 | 0 | 0 | 0 | 0 | 0 | 0 | 0 | 0 | 0 | 40 |
| Aquificota | 0 | 116 | 37 | 44 | 37 | 0 | 0 | 0 | 0 | 0 | 0 | 0 | 0 | 0 | 0 | 0 | 0 | 0 | 0 |
| Margulisbacteria | 0 | 83 | 103 | 64 | 42 | 32 | 41 | 90 | 0 | 0 | 0 | 0 | 0 | 0 | 0 | 0 | 0 | 0 | 0 |
| Riflebacteria | 0 | 43 | 0 | 0 | 0 | 0 | 0 | 0 | 0 | 0 | 0 | 0 | 0 | 0 | 0 | 0 | 0 | 0 | 0 |
| SZUA-182 | 0 | 64 | 78 | 55 | 54 | 0 | 0 | 54 | 0 | 0 | 0 | 0 | 0 | 0 | 0 | 0 | 0 | 0 | 0 |
| Firmicutes_H | 0 | 61 | 0 | 57 | 0 | 0 | 0 | 0 | 0 | 0 | 0 | 0 | 0 | 0 | 0 | 0 | 0 | 0 | 0 |
| Deferribacterota | 0 | 44 | 0 | 0 | 0 | 0 | 0 | 29 | 0 | 0 | 0 | 0 | 0 | 0 | 0 | 0 | 0 | 0 | 0 |
| UBP6 | 0 | 42 | 0 | 0 | 0 | 0 | 0 | 0 | 0 | 0 | 0 | 0 | 0 | 0 | 0 | 0 | 0 | 0 | 0 |
| Methanobacteriota_B | 0 | 324 | 81 | 90 | 56 | 0 | 0 | 0 | 0 | 0 | 0 | 0 | 0 | 0 | 0 | 0 | 0 | 0 | 0 |
| Methanobacteriota_A | 0 | 72 | 0 | 0 | 0 | 0 | 0 | 0 | 0 | 0 | 0 | 0 | 0 | 0 | 0 | 0 | 0 | 0 | 0 |
| Poribacteria | 0 | 0 | 128 | 2 | 0 | 0 | 0 | 0 | 0 | 0 | 0 | 0 | 0 | 0 | 0 | 0 | 0 | 0 | 0 |
| Dependentiae | 0 | 0 | 108 | 78 | 0 | 0 | 0 | 0 | 0 | 0 | 0 | 0 | 0 | 0 | 0 | 0 | 0 | 0 | 0 |
| WOR-3 | 0 | 0 | 102 | 51 | 0 | 0 | 0 | 0 | 0 | 0 | 0 | 0 | 0 | 0 | 0 | 0 | 0 | 0 | 0 |
| Dictyoglomota | 0 | 0 | 33 | 0 | 0 | 0 | 0 | 35 | 0 | 0 | 0 | 0 | 0 | 0 | 0 | 0 | 0 | 0 | 0 |
| Firmicutes_D | 0 | 0 | 43 | 0 | 0 | 0 | 0 | 51 | 49 | 0 | 0 | 0 | 42 | 0 | 0 | 0 | 0 | 0 | 0 |
| Aenigmatarchaeota | 0 | 0 | 582 | 343 | 0 | 0 | 0 | 0 | 0 | 0 | 0 | 0 | 71 | 0 | 0 | 0 | 0 | 0 | 0 |
| HKB111 | 0 | 0 | 0 | 0 | 0 | 0 | 0 | 0 | 0 | 0 | 0 | 0 | 0 | 0 | 0 | 0 | 0 | 0 | 0 |
| Cloacimonadota | 0 | 0 | 0 | 0 | 0 | 0 | 0 | 20 | 0 | 0 | 0 | 0 | 0 | 0 | 0 | 0 | 0 | 0 | 0 |
| Desulfobacterota_D | 0 | 0 | 0 | 0 | 0 | 0 | 0 | 11 | 0 | 0 | 0 | 0 | 0 | 0 | 0 | 0 | 0 | 0 | 0 |
| Atribacterota | 0 | 0 | 0 | 0 | 0 | 0 | 0 | 0 | 42 | 0 | 0 | 0 | 0 | 0 | 0 | 0 | 0 | 0 | 0 |

**Supplementary S5 and Supplementary S6 were shown in EXCEL because the content is too large.**

**Supplementary S7.** The summary and classification of metagenome-assembled genomes (MAGs).

| MAGs id | Contigs number | Genome size（bp） | Longest contig（bp） | N50 （contigs）（bp） | Mean contig length（bp） | Completeness（%） | Contamination（%） | Taxonomy |
| --- | --- | --- | --- | --- | --- | --- | --- | --- |
| MAG41 | 14 | 1,303,624 | 335,295 | 131,516 | 93,116 | 100.00 | 0.00 | d__Bacteria;p__Actinomycetota;c__Coriobacteriia;o__Coriobacteriales;f__Atopobiaceae;g__RGIG4373;s__Unclassified |
| MAG33 | 41 | 2,066,976 | 274,189 | 90,605 | 50,414 | 100.00 | 0.00 | d__Bacteria;p__Actinomycetota;c__Coriobacteriia;o__Coriobacteriales;f__Eggerthellaceae;g__Slackia_A;s__Slackia_A piriformis |
| MAG93 | 56 | 4,824,057 | 383,291 | 167,924 | 86,144 | 99.96 | 0.50 | d__Bacteria;p__Pseudomonadota;c__Gammaproteobacteria;o__Enterobacterales;f__Enterobacteriaceae;g__Escherichia;s__Escherichia coli |
| MAG123 | 213 | 4,730,408 | 185,519 | 38,194 | 22,208 | 99.76 | 0.57 | d__Bacteria;p__Pseudomonadota;c__Gammaproteobacteria;o__Enterobacterales;f__Enterobacteriaceae;g__Escherichia;s__Escherichia coli |
| MAG89 | 85 | 3,276,521 | 160,131 | 75,905 | 38,547 | 99.44 | 0.56 | d__Bacteria;p__Pseudomonadota;c__Gammaproteobacteria;o__Enterobacterales;f__Enterobacteriaceae;g__Plesiomonas;s__Plesiomonas shigelloides |
| MAG2 | 106 | 1,752,269 | 145,511 | 29,423 | 16,531 | 99.17 | 0.00 | d__Bacteria;p__Actinomycetota;c__Actinomycetia;o__Actinomycetales;f__Bifidobacteriaceae;g__Unclassified;s__Unclassified |
| MAG103 | 121 | 3,928,487 | 307,322 | 60,082 | 32,467 | 98.82 | 0.00 | d__Bacteria;p__Desulfobacterota;c__Desulfovibrionia;o__Desulfovibrionales;f__Desulfovibrionaceae;g__Bilophila;s__Bilophila wadsworthia |
| MAG126 | 425 | 3,306,786 | 73,709 | 11,162 | 7,781 | 98.31 | 0.85 | d__Bacteria;p__Pseudomonadota;c__Gammaproteobacteria;o__Enterobacterales;f__Enterobacteriaceae;g__Plesiomonas;s__Plesiomonas shigelloides |
| MAG85 | 80 | 4,429,088 | 406,716 | 92,034 | 55,364 | 98.12 | 1.15 | d__Bacteria;p__Bacteroidota;c__Bacteroidia;o__Bacteroidales;f__Bacteroidaceae;g__Phocaeicola;s__Phocaeicola vulgatus |
| MAG3 | 20 | 1,583,925 | 243,940 | 123,433 | 79,196 | 97.99 | 0.00 | d__Bacteria;p__Bacillota_A;c__Clostridia;o__Oscillospirales;f__Ruminococcaceae;g__Massiliimalia;s__Unclassified |
| MAG98 | 49 | 813,291 | 108,079 | 27,296 | 16,598 | 97.95 | 0.91 | d__Bacteria;p__Bacillota;c__Bacilli;o__Mycoplasmatales;f__Mycoplasmoidaceae;g__Unclassified;s__Unclassified |
| MAG25 | 54 | 1,677,493 | 121,891 | 47,861 | 31,065 | 97.90 | 0.70 | d__Bacteria;p__Bacillota_A;c__Clostridia;o__Tissierellales;f__Peptoniphilaceae;g__Anaerosphaera;s__Unclassified |
| MAG9 | 138 | 2,603,430 | 73,764 | 30,198 | 18,865 | 97.68 | 0.88 | d__Bacteria;p__Bacteroidota;c__Bacteroidia;o__Flavobacteriales;f__Flavobacteriaceae;g__Flavobacterium;s__Unclassified |
| MAG82 | 38 | 1,367,552 | 174,573 | 69,554 | 35,988 | 97.28 | 0.00 | d__Bacteria;p__Actinomycetota;c__Actinomycetia;o__Actinomycetales;f__Bifidobacteriaceae;g__Alloscardovia;s__Unclassified |
| MAG16 | 49 | 1,444,836 | 118,670 | 47,025 | 29,486 | 97.22 | 0.95 | d__Bacteria;p__Actinomycetota;c__Actinomycetia;o__Actinomycetales;f__Actinomycetaceae;g__Varibaculum;s__Unclassified |
| MAG17 | 114 | 2,258,434 | 88,345 | 32,349 | 19,811 | 96.85 | 3.15 | d__Bacteria;p__Bacillota_A;c__Clostridia;o__Peptostreptococcales;f__Peptostreptococcaceae;g__Peptostreptococcus;s__Unclassified |
| MAG6 | 476 | 4,036,457 | 163,161 | 43,006 | 8,480 | 96.28 | 9.57 | d__Bacteria;p__Bacteroidota;c__Bacteroidia;o__Bacteroidales;f__UBA7332;g__UBA7332;s__Unclassified |
| MAG61 | 532 | 4,662,880 | 127,806 | 19,594 | 8,765 | 96.09 | 2.54 | d__Bacteria;p__Pseudomonadota;c__Gammaproteobacteria;o__Enterobacterales;f__Enterobacteriaceae;g__Escherichia;s__Escherichia coli |
| MAG15 | 130 | 1,366,824 | 49,233 | 17,224 | 10,514 | 95.83 | 1.35 | d__Bacteria;p__Campylobacterota;c__Campylobacteria;o__Campylobacterales;f__Helicobacteraceae;g__Helicobacter_G;s__Unclassified |
| MAG68 | 56 | 1,446,362 | 110,762 | 56,250 | 25,828 | 95.81 | 1.11 | d__Bacteria;p__Actinomycetota;c__Actinomycetia;o__Actinomycetales;f__Actinomycetaceae;g__Varibaculum;s__Unclassified |
| MAG11 | 35 | 2,157,977 | 171,757 | 87,271 | 61,656 | 95.51 | 0.00 | d__Bacteria;p__Fusobacteriota;c__Fusobacteriia;o__Fusobacteriales;f__Fusobacteriaceae;g__Fusobacterium_B;s__Fusobacterium_B sp900541465 |
| MAG114 | 418 | 2,574,546 | 33,906 | 9,300 | 6,159 | 95.50 | 1.37 | d__Bacteria;p__Actinomycetota;c__Actinomycetia;o__Actinomycetales;f__Micrococcaceae;g__Kocuria;s__Kocuria tytonis |
| MAG101 | 471 | 4,230,848 | 38,534 | 12,686 | 8,983 | 95.29 | 1.71 | d__Bacteria;p__Pseudomonadota;c__Gammaproteobacteria;o__Enterobacterales;f__Enterobacteriaceae;g__Escherichia;s__Escherichia coli |
| MAG31 | 139 | 1,449,763 | 63,353 | 19,268 | 10,430 | 95.16 | 0.17 | d__Bacteria;p__Campylobacterota;c__Campylobacteria;o__Campylobacterales;f__Helicobacteraceae;g__Helicobacter_G;s__Unclassified |
| MAG63 | 302 | 1,106,960 | 17,670 | 4,856 | 3,665 | 95.00 | 1.61 | d__Bacteria;p__Actinomycetota;c__Coriobacteriia;o__Coriobacteriales;f__Atopobiaceae;g__CAJOGH01;s__Unclassified |
| MAG120 | 58 | 1,969,974 | 156,499 | 76,351 | 33,965 | 94.97 | 1.34 | d__Bacteria;p__Bacillota_A;c__Clostridia;o__Lachnospirales;f__Cellulosilyticaceae;g__Zhenhengia;s__Unclassified |
| MAG59 | 18 | 1,200,617 | 203,472 | 128,066 | 66,701 | 94.92 | 0.00 | d__Bacteria;p__Pseudomonadota;c__Gammaproteobacteria;o__Enterobacterales_A;f__Pasteurellaceae;g__Actinobacillus_C;s__Unclassified |
| MAG86 | 240 | 2,331,123 | 87,201 | 14,038 | 9,713 | 94.43 | 7.69 | d__Bacteria;p__Bacillota_A;c__Clostridia;o__Peptostreptococcales;f__Peptostreptococcaceae;g__Peptostreptococcus;s__Unclassified |
| MAG23 | 384 | 2,311,295 | 77,700 | 10,688 | 6,019 | 94.18 | 1.13 | d__Bacteria;p__Actinomycetota;c__Actinomycetia;o__Mycobacteriales;f__Mycobacteriaceae;g__Corynebacterium;s__Unclassified |
| MAG106 | 34 | 1,439,231 | 238,269 | 120,365 | 42,330 | 94.07 | 0.35 | d__Bacteria;p__Campylobacterota;c__Campylobacteria;o__Campylobacterales;f__Helicobacteraceae;g__Helicobacter_G;s__Unclassified |
| MAG54 | 37 | 1,266,417 | 177,313 | 72,409 | 34,227 | 93.72 | 1.05 | d__Bacteria;p__Bacillota;c__Bacilli;o__Lactobacillales;f__Lactobacillaceae;g__F6-6636;s__Unclassified |
| MAG79 | 67 | 2,478,671 | 151,338 | 61,722 | 36,995 | 93.55 | 0.81 | d__Bacteria;p__Actinomycetota;c__Coriobacteriia;o__Coriobacteriales;f__Eggerthellaceae;g__Paraeggerthella;s__Paraeggerthella hongkongensis_A |
| MAG95 | 832 | 4,125,986 | 39,221 | 7,741 | 4,959 | 93.09 | 9.19 | d__Bacteria;p__Bacteroidota;c__Bacteroidia;o__Chitinophagales;f__Chitinophagaceae;g__Flavisolibacter;s__Unclassified |
| MAG115 | 23 | 1,156,019 | 227,846 | 129,017 | 50,262 | 92.67 | 1.57 | d__Bacteria;p__Bacillota;c__Bacilli;o__Lactobacillales;f__Lactobacillaceae;g__F6-6636;s__Unclassified |
| MAG111 | 618 | 2,099,815 | 20,721 | 4,420 | 3,398 | 92.61 | 2.22 | d__Bacteria;p__Bacillota_A;c__Clostridia;o__Lachnospirales;f__Lachnospiraceae;g__Ruminococcus_B;s__Unclassified |
| MAG52 | 841 | 3,036,673 | 36,230 | 4,890 | 3,611 | 92.34 | 4.05 | d__Bacteria;p__Pseudomonadota;c__Gammaproteobacteria;o__Enterobacterales;f__Enterobacteriaceae;g__Plesiomonas;s__Plesiomonas shigelloides |
| MAG67 | 200 | 1,828,005 | 63,347 | 16,746 | 9,140 | 92.33 | 0.47 | d__Bacteria;p__Bacillota_A;c__Clostridia;o__Lachnospirales;f__Lachnospiraceae;g__Blautia_A;s__Unclassified |
| MAG58 | 57 | 1,270,598 | 127,860 | 40,801 | 22,291 | 92.15 | 2.20 | d__Bacteria;p__Bacillota;c__Bacilli;o__Lactobacillales;f__Lactobacillaceae;g__F6-6636;s__Unclassified |
| MAG87 | 25 | 786,382 | 118,799 | 45,704 | 31,455 | 91.77 | 0.77 | d__Bacteria;p__Bacillota;c__Bacilli;o__Mycoplasmatales;f__Mycoplasmoidaceae;g__Ureaplasma;s__Unclassified |
| MAG122 | 113 | 1,430,530 | 77,082 | 18,882 | 12,660 | 91.61 | 1.62 | d__Bacteria;p__Bacillota;c__Bacilli;o__Lactobacillales;f__Lactobacillaceae;g__Ligilactobacillus;s__Unclassified |
| MAG64 | 860 | 4,015,181 | 23,640 | 5,729 | 4,669 | 91.49 | 3.25 | d__Bacteria;p__Pseudomonadota;c__Gammaproteobacteria;o__Enterobacterales;f__Enterobacteriaceae;g__Escherichia;s__Escherichia coli |
| MAG130 | 878 | 2,768,928 | 23,022 | 3,972 | 3,154 | 91.34 | 3.77 | d__Bacteria;p__Pseudomonadota;c__Gammaproteobacteria;o__Enterobacterales;f__Enterobacteriaceae;g__Plesiomonas;s__Plesiomonas shigelloides |
| MAG97 | 384 | 1,533,732 | 28,492 | 6,504 | 3,994 | 90.83 | 2.87 | d__Bacteria;p__Bacillota_A;c__Clostridia;o__Oscillospirales;f__Acutalibacteraceae;g__Unclassified;s__Unclassified |
| MAG26 | 316 | 1,915,839 | 38,274 | 10,102 | 6,063 | 90.75 | 7.46 | d__Bacteria;p__Bacillota;c__Bacilli;o__Lactobacillales;f__Lactobacillaceae;g__Lactobacillus;s__Lactobacillus crispatus |
| MAG30 | 688 | 2,120,816 | 21,787 | 4,048 | 3,083 | 90.63 | 5.91 | d__Bacteria;p__Bacillota_A;c__Clostridia;o__Lachnospirales;f__Lachnospiraceae;g__Ruminococcus_B;s__Unclassified |
| MAG78 | 447 | 3,625,369 | 52,704 | 10,506 | 8,110 | 90.09 | 1.48 | d__Bacteria;p__Bacteroidota;c__Bacteroidia;o__Chitinophagales;f__Chitinophagaceae;g__Segetibacter;s__Unclassified |
| MAG80 | 370 | 1,623,458 | 26,308 | 6,404 | 4,388 | 89.52 | 2.98 | d__Bacteria;p__Bacillota;c__Bacilli;o__Lactobacillales;f__Streptococcaceae;g__Streptococcus;s__Streptococcus equinus |
| MAG117 | 73 | 3,987,429 | 351,232 | 105,071 | 54,622 | 89.16 | 0.96 | d__Bacteria;p__Bacteroidota;c__Bacteroidia;o__Sphingobacteriales;f__Sphingobacteriaceae;g__Pedobacter;s__Unclassified |
| MAG60 | 55 | 2,719,572 | 241,262 | 91,818 | 49,447 | 88.76 | 1.12 | d__Bacteria;p__Fusobacteriota;c__Fusobacteriia;o__Fusobacteriales;f__Fusobacteriaceae;g__Cetobacterium_A;s__Unclassified |
| MAG57 | 317 | 3,231,223 | 78,167 | 21,223 | 10,193 | 88.64 | 2.68 | d__Bacteria;p__Pseudomonadota;c__Gammaproteobacteria;o__Enterobacterales;f__Enterobacteriaceae;g__Edwardsiella;s__Edwardsiella hoshinae |
| MAG56 | 955 | 2,958,481 | 30,850 | 3,763 | 3,098 | 88.61 | 6.21 | d__Bacteria;p__Pseudomonadota;c__Gammaproteobacteria;o__Enterobacterales;f__Enterobacteriaceae;g__Plesiomonas;s__Plesiomonas shigelloides |
| MAG71 | 82 | 1,672,346 | 83,423 | 38,451 | 20,394 | 88.11 | 3.50 | d__Bacteria;p__Bacillota_A;c__Clostridia;o__Tissierellales;f__Peptoniphilaceae;g__Anaerosphaera;s__Unclassified |
| MAG128 | 245 | 1,430,970 | 38,467 | 7,831 | 5,841 | 87.59 | 4.22 | d__Bacteria;p__Bacillota_A;c__Clostridia;o__Peptostreptococcales;f__Anaerovoracaceae;g__Unclassified;s__Unclassified |
| MAG49 | 691 | 1,892,162 | 12,484 | 3,326 | 2,738 | 86.78 | 4.21 | d__Bacteria;p__Actinomycetota;c__Actinomycetia;o__Actinomycetales;f__Actinomycetaceae;g__Actinomyces;s__Unclassified |
| MAG53 | 1,014 | 2,492,803 | 16,048 | 2,889 | 2,458 | 86.72 | 9.33 | d__Bacteria;p__Actinomycetota;c__Actinomycetia;o__Mycobacteriales;f__Mycobacteriaceae;g__Corynebacterium;s__Unclassified |
| MAG55 | 38 | 1,462,163 | 159,341 | 84,098 | 38,478 | 86.20 | 0.63 | d__Bacteria;p__Actinomycetota;c__Actinomycetia;o__Actinomycetales;f__Actinomycetaceae;g__Varibaculum;s__Unclassified |
| MAG45 | 968 | 3,998,067 | 25,849 | 4,944 | 4,130 | 86.20 | 4.96 | d__Bacteria;p__Pseudomonadota;c__Gammaproteobacteria;o__Enterobacterales;f__Enterobacteriaceae;g__Escherichia;s__Escherichia coli |
| MAG24 | 916 | 4,199,387 | 29,344 | 5,588 | 4,584 | 83.30 | 8.69 | d__Bacteria;p__Bacteroidota;c__Bacteroidia;o__Sphingobacteriales;f__Sphingobacteriaceae;g__Pedobacter;s__Unclassified |
| MAG62 | 1,402 | 3,533,762 | 18,637 | 2,917 | 2,521 | 83.16 | 4.51 | d__Bacteria;p__Pseudomonadota;c__Gammaproteobacteria;o__Enterobacterales;f__Enterobacteriaceae;g__Escherichia;s__Escherichia coli |
| MAG124 | 146 | 3,725,588 | 209,960 | 54,914 | 25,518 | 82.93 | 3.45 | d__Bacteria;p__Bacillota_A;c__Clostridia;o__Clostridiales;f__Clostridiaceae;g__Clostridium_J;s__Clostridium_J culturomicium |
| MAG96 | 149 | 1,317,633 | 39,942 | 12,199 | 8,843 | 82.85 | 0.78 | d__Bacteria;p__Bacillota;c__Bacilli;o__Lactobacillales;f__Streptococcaceae;g__Streptococcus;s__Unclassified |
| MAG110 | 34 | 2,248,204 | 331,353 | 174,448 | 66,124 | 82.76 | 1.72 | d__Bacteria;p__Bacillota_A;c__Clostridia;o__Clostridiales;f__Clostridiaceae;g__Clostridium_J;s__Unclassified |
| MAG69 | 496 | 1,549,736 | 24,299 | 3,750 | 3,124 | 82.15 | 1.43 | d__Bacteria;p__Bacillota;c__Bacilli;o__Staphylococcales;f__Staphylococcaceae;g__Macrococcus_B;s__Macrococcus_B sp019357535 |
| MAG18 | 789 | 1,939,936 | 17,397 | 2,703 | 2,459 | 81.91 | 0.00 | d__Bacteria;p__Bacillota;c__Bacilli;o__Erysipelotrichales;f__Erysipelotrichaceae;g__Amedibacterium;s__Unclassified |
| MAG20 | 186 | 1,201,261 | 27,134 | 7,984 | 6,458 | 81.44 | 1.42 | d__Bacteria;p__Bacillota_A;c__Clostridia;o__Peptostreptococcales;f__Anaerovoracaceae;g__Unclassified;s__Unclassified |
| MAG34 | 1,421 | 3,341,170 | 13,427 | 2,604 | 2,351 | 80.49 | 5.67 | d__Bacteria;p__Pseudomonadota;c__Gammaproteobacteria;o__Burkholderiales;f__Burkholderiaceae_B;g__Ramlibacter;s__Unclassified |
| MAG13 | 494 | 1,733,302 | 16,690 | 4,634 | 3,509 | 80.33 | 4.13 | d__Bacteria;p__Actinomycetota;c__Actinomycetia;o__Mycobacteriales;f__Mycobacteriaceae;g__Corynebacterium;s__Unclassified |
| MAG14 | 194 | 1,326,324 | 30,404 | 8,993 | 6,837 | 79.56 | 1.11 | d__Bacteria;p__Bacillota_A;c__Clostridia;o__Peptostreptococcales;f__Anaerovoracaceae;g__Unclassified;s__Unclassified |
| MAG39 | 1,569 | 3,481,465 | 11,907 | 2,542 | 2,219 | 79.33 | 9.46 | d__Bacteria;p__Pseudomonadota;c__Gammaproteobacteria;o__Enterobacterales;f__Enterobacteriaceae;g__Escherichia;s__Escherichia coli |
| MAG84 | 163 | 2,598,644 | 141,642 | 45,131 | 15,943 | 79.31 | 3.45 | d__Bacteria;p__Bacillota_A;c__Clostridia;o__Clostridiales;f__Clostridiaceae;g__Hathewaya;s__Unclassified |
| MAG42 | 157 | 2,868,182 | 125,425 | 59,204 | 18,269 | 79.31 | 0.00 | d__Bacteria;p__Bacillota_A;c__Clostridia;o__Peptostreptococcales;f__Peptostreptococcaceae;g__Paraclostridium;s__Unclassified |
| MAG112 | 1,082 | 3,236,389 | 29,307 | 3,658 | 2,991 | 79.24 | 3.80 | d__Bacteria;p__Pseudomonadota;c__Gammaproteobacteria;o__Enterobacterales;f__Enterobacteriaceae;g__Escherichia;s__Escherichia coli |
| MAG90 | 38 | 1,521,260 | 233,483 | 107,167 | 40,033 | 79.17 | 0.00 | d__Bacteria;p__Bacillota_B;c__Peptococcia;o__Peptococcales;f__Peptococcaceae;g__Unclassified;s__Unclassified |
| MAG75 | 496 | 2,130,957 | 40,006 | 6,335 | 4,296 | 78.53 | 1.51 | d__Bacteria;p__Bacteroidota;c__Bacteroidia;o__Bacteroidales;f__UBA7332;g__UBA7332;s__UBA7332 sp002471225 |
| MAG88 | 94 | 2,093,116 | 129,689 | 49,740 | 22,267 | 77.78 | 2.37 | d__Bacteria;p__Bacillota_A;c__Clostridia;o__Clostridiales;f__Clostridiaceae;g__Clostridium;s__Clostridium moniliforme |
| MAG109 | 237 | 1,017,962 | 20,763 | 4,696 | 4,295 | 77.37 | 1.35 | d__Bacteria;p__Campylobacterota;c__Campylobacteria;o__Campylobacterales;f__Helicobacteraceae;g__Helicobacter_G;s__Unclassified |
| MAG113 | 400 | 976,941 | 7,830 | 2,784 | 2,442 | 77.23 | 5.33 | d__Bacteria;p__Bacillota;c__Bacilli;o__Lactobacillales;f__Lactobacillaceae;g__F6-6636;s__Unclassified |
| MAG7 | 369 | 1,128,795 | 17,366 | 3,633 | 3,059 | 76.53 | 3.33 | d__Bacteria;p__Actinomycetota;c__Actinomycetia;o__Actinomycetales;f__Actinomycetaceae;g__ZJ293;s__Unclassified |
| MAG47 | 648 | 1,610,381 | 10,754 | 2,828 | 2,485 | 76.39 | 3.35 | d__Bacteria;p__Bacteroidota;c__Bacteroidia;o__Flavobacteriales;f__Weeksellaceae;g__Kaistella;s__Unclassified |
| MAG73 | 959 | 4,391,555 | 20,548 | 5,353 | 4,579 | 76.30 | 1.29 | d__Bacteria;p__Bacteroidota;c__Bacteroidia;o__Cytophagales;f__Spirosomaceae;g__Dyadobacter;s__Unclassified |
| MAG105 | 554 | 1,851,580 | 20,019 | 4,488 | 3,342 | 76.26 | 6.80 | d__Bacteria;p__Actinomycetota;c__Actinomycetia;o__Actinomycetales;f__Dermabacteraceae;g__TAE3-ERU5;s__Unclassified |
| MAG27 | 745 | 2,388,527 | 19,587 | 3,472 | 3,206 | 75.44 | 5.62 | d__Bacteria;p__Pseudomonadota;c__Alphaproteobacteria;o__Sphingomonadales;f__Sphingomonadaceae;g__Croceibacterium;s__Unclassified |
| MAG12 | 556 | 1,200,662 | 10,819 | 2,332 | 2,159 | 75.15 | 5.42 | d__Bacteria;p__Campylobacterota;c__Campylobacteria;o__Campylobacterales;f__Helicobacteraceae;g__Helicobacter_G;s__Unclassified |
| MAG94 | 506 | 1,797,135 | 19,553 | 4,183 | 3,552 | 74.79 | 1.69 | d__Bacteria;p__Fusobacteriota;c__Fusobacteriia;o__Fusobacteriales;f__Fusobacteriaceae;g__Cetobacterium_A;s__Unclassified |
| MAG38 | 334 | 1,625,539 | 33,525 | 8,141 | 4,867 | 72.91 | 7.57 | d__Bacteria;p__Actinomycetota;c__Actinomycetia;o__Actinomycetales;f__Actinomycetaceae;g__Boudabousia;s__Unclassified |
| MAG81 | 1,093 | 2,340,676 | 33,567 | 2,315 | 2,142 | 71.94 | 3.15 | d__Bacteria;p__Pseudomonadota;c__Gammaproteobacteria;o__Enterobacterales;f__Enterobacteriaceae;g__Plesiomonas;s__Plesiomonas shigelloides |
| MAG19 | 494 | 1,620,535 | 20,730 | 4,567 | 3,280 | 71.84 | 1.46 | d__Bacteria;p__Bacillota_A;c__Clostridia;o__Lachnospirales;f__Lachnospiraceae;g__Ruminococcus_B;s__Unclassified |
| MAG72 | 96 | 1,899,871 | 108,625 | 29,965 | 19,790 | 71.55 | 1.72 | d__Bacteria;p__Fusobacteriota;c__Fusobacteriia;o__Fusobacteriales;f__Fusobacteriaceae;g__Fusobacterium_A;s__Fusobacterium_A sp900543175 |
| MAG74 | 54 | 1,433,999 | 143,104 | 50,218 | 26,556 | 70.69 | 1.72 | d__Bacteria;p__Fusobacteriota;c__Fusobacteriia;o__Fusobacteriales;f__Fusobacteriaceae;g__Fusobacterium_A;s__Unclassified |
| MAG35 | 50 | 1,760,754 | 151,172 | 96,066 | 35,215 | 70.69 | 0.00 | d__Bacteria;p__Bacillota_A;c__Clostridia;o__Clostridiales;f__Clostridiaceae;g__Sarcina;s__Unclassified |
| MAG22 | 339 | 1,181,772 | 16,055 | 3,950 | 3,486 | 69.54 | 6.93 | d__Bacteria;p__Bacillota_A;c__Clostridia;o__Peptostreptococcales;f__Anaerovoracaceae;g__Unclassified;s__Unclassified |
| MAG5 | 1,429 | 3,468,136 | 24,732 | 2,796 | 2,427 | 68.97 | 3.45 | d__Bacteria;p__Pseudomonadota;c__Gammaproteobacteria;o__Enterobacterales;f__Enterobacteriaceae;g__Escherichia;s__Escherichia coli |
| MAG37 | 663 | 2,426,474 | 18,867 | 3,917 | 3,660 | 68.04 | 3.86 | d__Bacteria;p__Bacteroidota;c__Bacteroidia;o__Sphingobacteriales;f__Sphingobacteriaceae;g__Daejeonella;s__Unclassified |
| MAG107 | 98 | 621,939 | 70,199 | 9,122 | 6,346 | 66.92 | 5.11 | d__Bacteria;p__Bacillota;c__Bacilli;o__Mycoplasmatales;f__UBA3375;g__UBA3375;s__Unclassified |
| MAG36 | 234 | 1,187,639 | 30,902 | 6,591 | 5,075 | 66.90 | 0.53 | d__Bacteria;p__Bacillota_B;c__Peptococcia;o__Peptococcales;f__Peptococcaceae;g__Unclassified;s__Unclassified |
| MAG32 | 383 | 821,500 | 9,063 | 2,341 | 2,145 | 66.46 | 2.61 | d__Bacteria;p__Bacillota;c__Bacilli;o__Lactobacillales;f__Lactobacillaceae;g__F6-6636;s__Unclassified |
| MAG125 | 45 | 2,760,228 | 520,560 | 147,070 | 61,338 | 65.41 | 0.38 | d__Bacteria;p__Bacteroidota;c__Bacteroidia;o__Bacteroidales;f__Unclassified;g__Unclassified;s__Unclassified |
| MAG108 | 143 | 815,100 | 24,533 | 7,647 | 5,700 | 64.35 | 1.75 | d__Bacteria;p__Bacillota;c__Bacilli;o__Mycoplasmatales;f__UBA3375;g__UBA3375;s__Unclassified |
| MAG66 | 337 | 1,023,331 | 15,517 | 3,737 | 3,037 | 64.19 | 1.74 | d__Bacteria;p__Bacillota;c__Bacilli;o__Lactobacillales;f__Lactobacillaceae;g__Limosilactobacillus;s__Limosilactobacillus reuteri_E |
| MAG121 | 772 | 1,607,941 | 13,692 | 2,189 | 2,083 | 64.09 | 1.84 | d__Bacteria;p__Verrucomicrobiota;c__Verrucomicrobiae;o__Verrucomicrobiales;f__Akkermansiaceae;g__Akkermansia;s__Akkermansia muciniphila |
| MAG40 | 55 | 2,205,717 | 241,239 | 121,263 | 40,104 | 63.35 | 1.35 | d__Bacteria;p__Pseudomonadota;c__Gammaproteobacteria;o__Burkholderiales;f__Burkholderiaceae_A;g__Sutterella;s__Unclassified |
| MAG50 | 219 | 2,586,765 | 58,512 | 19,665 | 11,812 | 63.21 | 3.54 | d__Bacteria;p__Bacillota_A;c__Clostridia;o__Clostridiales;f__Clostridiaceae;g__Clostridium;s__Unclassified |
| MAG116 | 1,330 | 2,503,136 | 8,937 | 1,948 | 1,882 | 62.96 | 1.14 | d__Bacteria;p__Pseudomonadota;c__Gammaproteobacteria;o__Enterobacterales;f__Enterobacteriaceae;g__Escherichia;s__Escherichia coli |
| MAG1 | 509 | 1,038,836 | 9,966 | 2,218 | 2,041 | 62.86 | 2.08 | d__Bacteria;p__Bacillota;c__Bacilli;o__Lactobacillales;f__Streptococcaceae;g__Streptococcus;s__Unclassified |
| MAG4 | 377 | 2,768,874 | 98,671 | 22,630 | 7,344 | 62.68 | 1.61 | d__Bacteria;p__Pseudomonadota;c__Gammaproteobacteria;o__Enterobacterales;f__Enterobacteriaceae;g__Edwardsiella;s__Edwardsiella tarda |
| MAG127 | 668 | 1,190,881 | 8,147 | 1,832 | 1,783 | 62.04 | 8.13 | d__Bacteria;p__Bacteroidota;c__Bacteroidia;o__Bacteroidales;f__Porphyromonadaceae;g__Porphyromonas;s__Unclassified |
| MAG10 | 679 | 1,335,328 | 7,885 | 2,105 | 1,967 | 61.76 | 1.60 | d__Bacteria;p__Bacillota_C;c__Negativicutes;o__Selenomonadales;f__Selenomonadaceae;g__Unclassified;s__Unclassified |
| MAG99 | 661 | 1,188,802 | 7,423 | 1,821 | 1,798 | 61.26 | 9.09 | d__Bacteria;p__Bacillota;c__Bacilli;o__Lactobacillales;f__Streptococcaceae;g__Lactococcus;s__Lactococcus formosensis |
| MAG28 | 189 | 1,594,932 | 44,225 | 11,052 | 8,439 | 61.13 | 3.45 | d__Bacteria;p__Fusobacteriota;c__Fusobacteriia;o__Fusobacteriales;f__Fusobacteriaceae;g__Fusobacterium_A;s__Unclassified |
| MAG76 | 349 | 1,199,411 | 26,230 | 5,155 | 3,437 | 60.63 | 3.89 | d__Bacteria;p__Patescibacteria;c__Saccharimonadia;o__Saccharimonadales;f__UBA2112;g__UBA2112;s__Unclassified |
| MAG91 | 130 | 2,147,040 | 84,832 | 26,585 | 16,516 | 60.34 | 1.72 | d__Bacteria;p__Bacillota_A;c__Clostridia;o__Clostridiales;f__Clostridiaceae;g__Clostridium_AH;s__Clostridium_AH fallax |
| MAG51 | 202 | 604,249 | 10,445 | 3,416 | 2,991 | 59.53 | 1.44 | d__Bacteria;p__Bacillota;c__Bacilli;o__Mycoplasmatales;f__UBA3375;g__UBA3375;s__Unclassified |
| MAG8 | 624 | 1,886,533 | 10,415 | 3,205 | 3,023 | 59.48 | 0.00 | d__Bacteria;p__Bacteroidota;c__Bacteroidia;o__Flavobacteriales;f__Flavobacteriaceae;g__Flavobacterium;s__Unclassified |
| MAG48 | 81 | 632,464 | 28,276 | 9,810 | 7,808 | 57.77 | 0.00 | d__Bacteria;p__Campylobacterota;c__Campylobacteria;o__Campylobacterales;f__Campylobacteraceae;g__Campylobacter_B;s__Unclassified |
| MAG70 | 887 | 1,593,363 | 8,479 | 1,845 | 1,796 | 57.52 | 3.75 | d__Bacteria;p__Bacillota_A;c__Clostridia;o__Clostridiales;f__Clostridiaceae;g__Sarcina;s__Sarcina perfringens |
| MAG83 | 241 | 864,600 | 33,975 | 4,707 | 3,588 | 57.19 | 0.16 | d__Bacteria;p__Fusobacteriota;c__Fusobacteriia;o__Fusobacteriales;f__Fusobacteriaceae;g__Fusobacterium_A;s__Unclassified |
| MAG21 | 629 | 1,077,777 | 10,562 | 1,723 | 1,713 | 56.11 | 9.43 | d__Bacteria;p__Bacillota_C;c__Negativicutes;o__Veillonellales;f__Veillonellaceae;g__Unclassified;s__Unclassified |
| MAG77 | 430 | 1,060,831 | 14,587 | 2,682 | 2,467 | 55.43 | 2.01 | d__Bacteria;p__Bacillota_A;c__Clostridia;o__Oscillospirales;f__Ruminococcaceae;g__Massiliimalia;s__Unclassified |
| MAG29 | 789 | 1,527,801 | 9,867 | 1,975 | 1,936 | 54.81 | 1.85 | d__Bacteria;p__Bacillota_A;c__Clostridia;o__Lachnospirales;f__Lachnospiraceae;g__Ruminococcus_B;s__Unclassified |
| MAG100 | 172 | 443,502 | 9,249 | 3,155 | 2,579 | 54.31 | 0.00 | d__Bacteria;p__Bacillota_A;c__Clostridia;o__Peptostreptococcales;f__Peptostreptococcaceae;g__GCA-900066495;s__Unclassified |
| MAG118 | 1,326 | 1,620,845 | 4,192 | 1,215 | 1,222 | 54.26 | 7.76 | d__Bacteria;p__Pseudomonadota;c__Alphaproteobacteria;o__Caulobacterales;f__Caulobacteraceae;g__Unclassified;s__Unclassified |
| MAG44 | 1,083 | 2,076,840 | 10,080 | 1,977 | 1,918 | 54.13 | 2.06 | d__Bacteria;p__Pseudomonadota;c__Gammaproteobacteria;o__Enterobacterales;f__Enterobacteriaceae;g__Edwardsiella;s__Edwardsiella hoshinae |
| MAG119 | 131 | 695,238 | 30,453 | 8,312 | 5,307 | 53.63 | 1.12 | d__Bacteria;p__Fusobacteriota;c__Fusobacteriia;o__Fusobacteriales;f__Fusobacteriaceae;g__Fusobacterium_A;s__Unclassified |
| MAG65 | 349 | 589,129 | 6,589 | 1,722 | 1,688 | 52.82 | 0.81 | d__Bacteria;p__Actinomycetota;c__Coriobacteriia;o__Coriobacteriales;f__Atopobiaceae;g__CAJOGH01;s__Unclassified |
| MAG92 | 403 | 890,865 | 10,279 | 2,442 | 2,211 | 51.81 | 1.07 | d__Bacteria;p__Campylobacterota;c__Campylobacteria;o__Campylobacterales;f__Helicobacteraceae;g__Helicobacter_G;s__Unclassified |
| MAG104 | 493 | 1,585,210 | 24,404 | 3,762 | 3,215 | 51.75 | 7.02 | d__Bacteria;p__Campylobacterota;c__Campylobacteria;o__Campylobacterales;f__Helicobacteraceae;g__Helicobacter_G;s__Unclassified |
| MAG129 | 514 | 1,267,975 | 21,214 | 2,884 | 2,467 | 51.72 | 3.45 | d__Bacteria;p__Bacillota_C;c__Negativicutes;o__Selenomonadales;f__Selenomonadaceae;g__Unclassified;s__Unclassified |
| MAG46 | 221 | 768,707 | 18,905 | 3,809 | 3,478 | 51.56 | 1.79 | d__Bacteria;p__Bacillota_C;c__Negativicutes;o__Veillonellales;f__Veillonellaceae;g__Unclassified;s__Unclassified |
| MAG43 | 436 | 709,791 | 8,199 | 1,642 | 1,628 | 51.43 | 1.90 | d__Bacteria;p__Actinomycetota;c__Coriobacteriia;o__Coriobacteriales;f__Atopobiaceae;g__RGIG4373;s__Unclassified |
| MAG102 | 186 | 2,155,732 | 74,865 | 20,337 | 11,590 | 50.31 | 0.62 | d__Bacteria;p__Pseudomonadota;c__Gammaproteobacteria;o__Burkholderiales;f__Burkholderiaceae_A;g__Sutterella;s__Sutterella sp905186105 |
